# A bacterial transcription activator dedicated to the expression of the enzyme catalyzing the first committed step in fatty acid biosynthesis

**DOI:** 10.1093/nar/gkae960

**Published:** 2024-10-30

**Authors:** Yuanyou Xu, Zihan Lin, Jiyuan Hou, Kai Ye, Sirui Han, Yuxuan Liang, Huihui Liang, Shihua Wu, Yizhi J Tao, Haichun Gao

**Affiliations:** Institute of Microbiology, College of Life Sciences, Zhejiang University, 866 Yuhangtang Road, Hangzhou, Zhejiang 310058, China; Department of BioSciences, Rice University, 6100 Main Street, Houston, TX, 77005, USA; Institute of Microbiology, College of Life Sciences, Zhejiang University, 866 Yuhangtang Road, Hangzhou, Zhejiang 310058, China; Department of BioSciences, Rice University, 6100 Main Street, Houston, TX, 77005, USA; Institute of Microbiology, College of Life Sciences, Zhejiang University, 866 Yuhangtang Road, Hangzhou, Zhejiang 310058, China; Institute of Microbiology, College of Life Sciences, Zhejiang University, 866 Yuhangtang Road, Hangzhou, Zhejiang 310058, China; Institute of Microbiology, College of Life Sciences, Zhejiang University, 866 Yuhangtang Road, Hangzhou, Zhejiang 310058, China; Institute of Microbiology, College of Life Sciences, Zhejiang University, 866 Yuhangtang Road, Hangzhou, Zhejiang 310058, China; Department of BioSciences, Rice University, 6100 Main Street, Houston, TX, 77005, USA; Institute of Microbiology, College of Life Sciences, Zhejiang University, 866 Yuhangtang Road, Hangzhou, Zhejiang 310058, China; Department of BioSciences, Rice University, 6100 Main Street, Houston, TX, 77005, USA

## Abstract

Acetyl-CoA carboxylase (ACCase) catalyzes the first committed and rate-limiting step of *de novo* fatty acid synthesis (FAS). Although this step is tightly regulated, regulators that specifically control transcription of the ACCase genes remain elusive. In this study, we identified LysR-type transcriptional regulator AccR as a dedicated activator for the transcription of *accS*, a gene encoding a multiple-domain ACCase in *Shewanella oneidensis*. We showed that AccR interacts with the *accS* promoter *in vivo* in response to changes in acetyl-CoA levels and *in vitro*. Analysis of the crystal structure of the effector-binding domain (EBD) of AccR identified two potential ligand-binding pockets, one of which is likely to bind acetyl-CoA as a ligand based on results from molecular docking, direct binding assay and mutational analysis of the residues predicted to interact with acetyl-CoA. Despite this, the interaction between AccR and acetyl-CoA alone appears unstable, implying that an additional yet unknown ligand is required for activation of AccR. Furthermore, we showed that AccR is acetylated, but the modification may not be critical for sensing acetyl-CoA. Overall, our data substantiate the existence of a dedicated transcriptional regulator for ACCases, expanding our current understanding of the regulation of FAS.

## Graphical Abstract

**Graphical Abstract:**
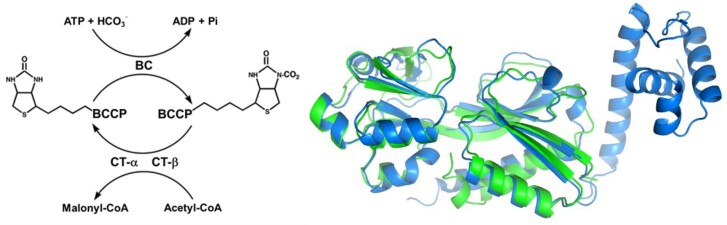

## Introduction

**Figure 1.**
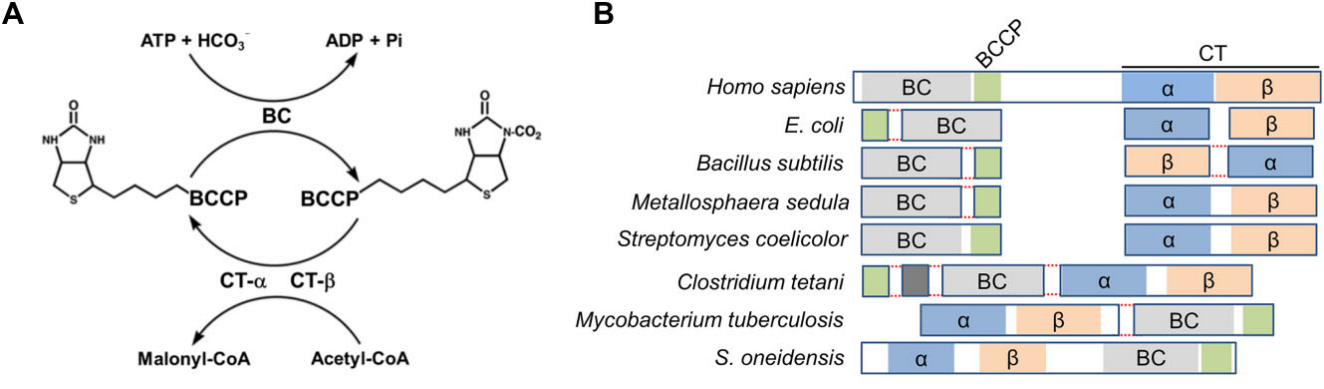
Reaction and subunit/domain organization of ACCases. (**A**) The reactions catalyzed by an ACCase. BC generates and attaches an activated carboxyl group covalently to biotin, which is linked covalently to BCCP, and CT transfers the carboxyl group to acetyl-CoA, producing malonyl-CoA. (**B**) Subunit/domain organization of ACCases from representative organisms. The same functional subunits and domains are shown in the same color. Multiple domains of a polypeptide are in rectangle of solid blue lines. Multiple subunits (polypeptides) linked by dashed red line indicate that their coding genes are clustered in the chromosomes, which may or may not be organized into a single operon.

*De novo* fatty acid synthesis (FAS) is one of the most fundamental biochemical processes in all domains of living organisms. The first committed step of this biosynthesis is the carboxylation of acetyl-CoA to produce malonyl-CoA, which is catalyzed by acetyl-CoA carboxylase (ACCase) (1,2) (Figure 1A). In most bacteria, ACCases have been well-characterized as a multi-subunit enzyme instead of a single polypeptide of multiple functional domains that are found in eukaryotes (3–5) (Figure 1B). Despite this difference, ACCases of both prokaryotes and eukaryotes contain three essential functional units: a homodimer of the biotin carboxylase (BC), the biotin carboxyl carrier protein (BCCP) and a heterotetramer α2β2 of the carboxyltransferase (CT) (2,3) (Figure 1A). BC catalyzes the first half-reaction, the ATP-dependent carboxylation of biotin, which is covalently linked to BCCP. The second half-reaction is catalyzed by CT, which transfers the carboxyl group from carboxybiotin to acetyl-CoA, generating malonyl-CoA (4).

Similar to other key metabolic enzymes, both bacterial and eukaryotic ACCases have been found to be subjected to feedback inhibition by downstream metabolites, including palmitoyl-CoA and acylated acyl-carrier protein (6–8). However, although transcription is the most decisive regulatory step for expression of metabolic enzymes, at present little is known about how transcription of *acc* genes responds to metabolic changes. In *Escherichia coli*, the genes coding for the BC (AccC) and BCCP (AccB) form a single operon but those for the CT-α (AccA) and CT-β (AccD) subunits are located elsewhere on the chromosome separately (Figure 1B). However, several different gene organizations have been observed in other bacteria, and an unusual example is *accS* of γ-proteobacterium *Shewanella oneidensis*, which encodes a multiple-domain ACCase (Figure 1B and Supplementary Figure S1) (1,4,5,9,10). Interestingly, this ACCase clearly differs from its eukaryotic counterparts in the order of the domains (Figure 1B).

Given that the overall reaction involves two separate half-reactions, it is not surprising that expression of the *acc* genes is coordinated in *E. coli* (11,12). AccB has been proposed to act as an autoregulator of *accBC* operon transcription in spite of the lack of a DNA-binding motif (13). An additional regulatory role is also projected for the CT subunits, as complexing CT with the *accA* and *accD* transcripts inhibited translation of these mRNAs and reduced CT activity (14), though this concept has been recently challenged (15). In a similar scenario, global regulator FadR (*f*atty *a*cid *d*egradation *r*egulator) was suggested to play a direct role in regulating all three operons for *E. coli* ACCase although the binding between FadR and the *accA* promoter *in vitro* was not demonstrated (16). The only confirmed transcriptional regulators that interact with the promoter regions of some, but not all, *acc* operons come from the TetR family, including FasR of *Corynebacterium glutamicum* and SAV5279 of *Streptomyces avermitilis* (17,18). However, as these two regulators repress a large number of genes by responding to a variety of acyl-CoA molecules, they appear to function to coordinate short-chain acyl CoA homeostasis in a general manner.

Here, we describe the discovery of a LysR type transcriptional regulator (LTTR), AccR (for ACCase regulator) in *S. oneidensis*, which directly and specifically activates transcription of the *accS* gene as a canonical LTTR (Figure 1B). We showed that the deletion or overexpression of *accR* compromises growth. The structure of the effector binding domain (EBD) reveals that AccR has a relatively large effector binding pocket. Multiple lines of evidence indicate that acetyl-CoA is one of the effectors. These lines include acetyl-CoA responsive regulation, direct interaction between acetyl-CoA and AccR *in vitro* revealed by isothermal calorimetry (ITC) and microscale thermophoresis (MST) measurement, and *in silico* by molecular dynamics (MD) simulations. Although acetylation occurs at multiple residues of AccR, the modification overall is dispensable for sensing acetyl-CoA. Given that LTTRs comprise one of the largest families of transcriptional factors in prokaryotes, our findings underscore the presence of transcriptional regulator specifically controlling expression of ACCases.

## Materials and methods

### Bacterial strains, plasmids and culture conditions

The bacterial strains and plasmids used in this study are listed in Supplementary Table S1. Sequences of the primers used in this study are available upon request. All chemicals are from Sigma-Aldrich Co. unless otherwise noted. *E. coli* and *S. oneidensis* were grown aerobically in Lysogeny broth (LB, Difco, Detroit, MI) at 37 and 30°C for genetic manipulation. For specific purposes, defined media M9 (0.4% glucose) and MS, which is a PIPES-buffered minimal salts medium containing 30 mM lactate as electron donor (19), were also used for *E. coli* and *S. oneidensis*, respectively. When appropriate, the growth medium was supplemented with one or more of the following: 2,6-diaminopimelic acid (DAP), 0.3 mM; ampicillin, 50 μg/ml; kanamycin, 50 μg/ml; gentamicin, 15 μg/ml.

Growth of *E. coli* and *S. oneidensis* strains in liquid media under aerobic conditions was measured at 600 nm (OD_600_). Fresh media were inoculated with overnight cultures by 200-fold dilution (OD_600_, ∼0.01) and shaken at 200 rpm at 30°C. To assess growth differences between strains on LB agar plates, cells at the mid-exponential phase were adjusted to approximately 10^8^ colony forming units per ml, and followed by 10-fold serial dilutions. Ten microliters of each dilution was spotted onto plates, which were incubated at 30°C for 24 h before being read.

### In-frame mutant construction

In-frame deletion strains were constructed using the *att*-based fusion polymerase chain reaction (PCR) method as described previously (20). In brief, two fragments flanking the target gene were amplified by PCR and then linked by a second round of PCR. The fused fragments were introduced into plasmid pHGM01 using the Gateway BP clonase II enzyme mix (Invitrogen) according to the manufacturer’s instruction, maintained in the commonly-used Δ*dapA* conjugation donor strain *E. coli* WM3064, and subsequently transferred into *S. oneidensis* via conjugation. Transconjugants selected by resistance to gentamycin were subject to PCR for confirming integration of the mutagenized constructs into the chromosome. The correct ones were then grown in LB broth in the absence of NaCl and plated on LB supplemented with 10% sucrose. Gentamycin-sensitive and sucrose-resistant colonies were screened by PCR for deletions of the target genes. Mutants were verified by sequencing the region containing the intended mutations.

### Gene expression and site-directed mutagenesis

Plasmid pHGEN-Ptac, which employs IPTG-inducible promoter P*_tac_*, was used for expressing genes of interest (21). After verification by sequencing, the vectors were transferred into the relevant strains via conjugation. Genes of interest cloned into pHGEN-P*tac* were applied to site-directed mutagenesis with Quick-Change Kit (Agilent) according to the manufacturer’s guidelines. All substitutions were verified by DNA sequencing.

### Gene expression assay

Expression of target genes was estimated by their promoter activity assessed using a single-copy integrative *lacZ* reporter system as described previously (22). A fragment containing the sequence upstream of any target operon, covering predicted or determined promoter to the translation start codon, was generated by PCR and cloned into integrative reporter vector pHGEI01 and verified by sequencing. These plasmids were then transferred by conjugation into relevant *S. oneidensis* strains, allowing integration of the reporter construct into the chromosome and the antibiotic marker is then removed by an established approach (23). Cells grown to the mid-exponential phase were collected were harvested, washed with phosphate-buffered saline (PBS, pH 7.4), resuspended in the same buffer and sonicated with a Xinzhi Sonifier (JY92-IIDN, NingBo, China) at maximum output on ice until no whole bacterial cells were visible, and the protein extracts were collected after centrifugation at 3000 × *g* for 10 min. Protein concentrations of the cell lysates were determined by the bicinchoninic acid assay (Pierce Chemical), and β-galactosidase activity assays were performed with an assay kit from Solarbio (Beijing) according to the manufacturer’s instruction.

### SDS-PAGE and western blotting

Unless otherwise noted, protein extracts were prepared the same as for the expression assay. The extracts were mixed with 4X SDS-loading buffer, denatured for 10 min at 100°C and resolved by 12% sodiumdodecyl sulfate-polyacrylamide gel electrophoresis (SDS-PAGE) gel. The resolved proteins were either directly stained with Coomassie Brilliant Blue R-250 or subject to western blotting (WB). Protein transfer onto the polyvinylidene fluoride (PVDF) membrane (GE-Healthcare) was carried out for 1 h at 60 V in a Criterion blotter (Bio-Rad) with Tris-Glycine transfer buffer. The blotting membrane was probed with mouse anti-His_6_-tag or anti-acetylated lysine and goat anti-mouse IgG-HRP (1:100 000) as the primary and secondary antibodies (Beyotime), respectively. The blots were developed by chemiluminescence detection with SuperSignal West Dura Extended Duration Substrate kit (Invitrogen) and visualized with the CLiNX system.

### Protein expression and purification

DNA fragments encoding AccR variants generated by PCR were cloned into pET-28a and subsequently transformed into *E. coli* BL21(DE3) for expression of His_6_-tagged recombinant proteins. The BL21(DE3) cells were grown at 37°C to an OD_600_ of 0.5 in M9 minimal media, supplemented with lysine (100 mg), phenylalanine (100 mg), threonine (100 mg), isoleucine (50 mg), leucine (50 mg), valine (50 mg) and selenomethionine (60 mg), then induced with 1 mM isopropyl β-D-thiogalactoside (IPTG) and allowed to be expressed overnight at 15°C. Some of His_6_-tagged AccR variants were also expressed from pHGEN-P*tac* and purified from an *S. oneidensis accR* deletion strain (Δ*accR*). The Δ*accR* cells were grown at 30°C to an OD_600_ of 0.5 in LB, then induced with 1 mM IPTG and allowed to be expressed overnight at 15°C. Cells of *E. coli* and *S. oneidensis* were pelleted, washed with PBS (pH 7.4), and resuspended in 30 ml of lysis buffer (50 mM Tris [pH 8.0], 250 mM NaCl, 5 mM β-mercaptoethanol, 1 mM NaN_3_, 10% glycerol) and disrupted with a French press. Recombinant AccR variants in the clarified lysates were loaded onto His-Pur™ Ni-NTA resin (ThermoFisher) and eluted with 250 mM imidazole. The protein was then passed through size-exclusion chromatography using the HiLoad® 16/600 Superdex® 200 pg (GE) for further purification. Purified proteins were concentrated to 2.8 mg/ml in lysis buffer.

### Electrophoretic mobility shift assays (**EMSAs)**

To test interaction between AccR and promoter regions of its target genes, electrophoretic mobility shift assays (EMSAs) were conducted as previously described (24). DNA probes covering the predicted AccR binding sites were obtained by PCR, during which the double-stranded product was labelled with digoxigenin-ddUTP (Roche diagostics). The digoxigenin-labeled DNA probes were mixed with serial dilutions of purified AccR of varying concentrations in binding buffer (4 mM Tris-HCl [pH 8.0], 40 mM NaCl, 4 mM MgCl_2_, 4% glycerol) containing 0.75 μg of poly(dI-dC) at room temperature for 15 min. The DNA/protein mixtures were loaded on 7% native polyacrylamide gels for electrophoretic separation and the resulting gel was visualized with the CLiNX system.

### Verification and quantification of DNAs bound to AccR purified from *S. oneidensis*

AccR expressed and purified from *S. oneidensis* was bound with DNAs. After denaturing protein samples by heating for 10 min at 90°C, the DNAs were extracted with QIAquick PCR purification kit (Qiagen) and used as the template for PCR with primers targeting the *accS* promoter region. To determine the DNA region protected by AccR, the purified AccR samples were treated with DNase I and subjected to sequencing. For quantification, qPCR was performed with DNAs extracted from 100 μg purified AccR as the template and primers FP_3_ and RP, with genomic DNA used as the control. The analysis was carried out with an ABI7300 96-well qRT-PCR system (Applied Biosystems). The copy numbers of the DNA fragment from each protein sample were averaged from three replicates and normalized against that of the control.

### Mass spectrometry analysis

Purified AccR proteins were subjected to mass spectrometry as previously reported (25). Briefly, the protein (50 μg) was digested overnight with trypsin (Promega, Madison, WI) at a ratio of 30:1 (protein:enzyme, w/w, ∼200 units) in 50 mM ammonium bicarbonate at 37°C. The digests were analyzed by nanoLC–MS/MS using a QTOF Ultima hybrid quadrupole time-of-flight mass spectrometer (Q Exactive™ Plus, Thermo) coupled to an EASY-nLC 1000 UPLC system. The digests were injected onto a 5 mm × 300 μm i.d. Acclaim PepMap100 C18 μ-precolumn (Dionex/Thermo Scientific, Sunnyvale, CA) and separated on a 100 μm × 100 mm i.d. 1.7 μm BEH130 C18 column (Waters) using the following gradient conditions: 5–45% acetonitrile (ACN) in 0.2% formic acid in 35 min and 45–95% ACN in 5 min, 450 nl/min. MS/MS spectra were acquired on doubly, triply and quadruply charged ions, processed using Proteome Discoverer 1.3 Tandem mass spectra against the NCBInr database.

### Structure determination

Purified AccR EBD was concentrated to 2.8 mg/ml for initial crystal screening using commercially available crystallization screen kits. Diffraction quality crystals were obtained using hanging-drop vapor diffusion by mixing the protein solution with a well solution containing 27.5% PEG 4000 and 100 mM HEPES pH 7.0 at 1:1 ratio. Plate-shaped crystals appeared in 3–5 days after incubation at 20°C. Crystals were cryo-cooled in mother liquor with 10% additional glycerol. X-ray diffraction data were collected with native protein crystals and the Advance Photon Source, LS-CAT Beamline 21-ID-G (Supplementary Table S2). Raw diffraction intensity data were processed with HKL2000 software package (26). The resulting data were used for AutoSol wizard with PHENIX package (27,28). The structure was then iteratively refined with PHENIX and Coot (29) and all figures were created using PyMOL (30) http://www.pymol.org/.).

### Isothermal titration calorimetry (ITC)

ITC experiments were conducted on a MicroCal iTC_200_ (GE Healthcare) at 25°C. Acetyl-CoA solution and AccR were loaded into the injection syringe and sample cell respectively, both in HEPES buffer (pH 7.0) or Tris buffer (pH 7.0). Titrations were performed using 36 μmol of AccR and 1.2 mM of acetyl-CoA. The ITC run involved 27 injections of 10 μl of acetyl-CoA into the sample cell. Additionally, acetyl-CoA at the same concentration as the experimental group was injected into the blank buffer as a control group. Data were analyzed using Origin 7.0 software by fitting a titration curve to the corrected data using a single-site interaction model (MicroCal).

### Fluorescence polarization (FP) assays

Fluorescence polarization (FP) assays were conducted to determine the binding affinity of AccR for DNA in a binding buffer solution (100 mM KCl, 20 mM HEPES (N-2-hydroxyethylpiperazine-N-2-ethane sulfonic acid), 0.1 mM MgCl_2_, 1 mM TCEP (Tris(2-carboxyethyl)phosphine), pH 7.5). Purified AccR was titrated into the binding buffer solution that contains 1 nM 5′-fluoresceine-labeled *accS* promoter sequence and 1 μg of poly(dI-dC) DNA as non-specific competitor. Fluorescence anisotropy was measured using a Tecan Infinite® 200 PRO Microplate Reader, with excitation at 490 nm and emission at 530 nm. All measurements were taken in triplicate, and the binding and competition polarization data were analyzed using Origin 2021 software.

### Microscale thermophoresis (MST) measurement

Recombinant His_6_-tagged eGFP-AccR was expressed and purified from *E. coli* the same as for recombinant His_6_-tagged AccR. Binding affinity between acetyl-CoA and eGFP-AccR was measured using MST. The peak fractions of SEC were pooled and diluted to a final concentration of 15 nM using HEPES buffer, and supplemented with 0.05% Tween 20. A stock solution of 100 μM acetyl-CoA was prepared in the same buffer, and serial 2-fold dilutions were made to obtain a range of ligand concentrations. The protein sample and ligand were mixed in a 1:1 ratio and incubated at room temperature for 30 min. The mixture was then loaded into capillaries for the MST assay. The measurements were performed at 25°C using 20% LED power and 40% MST power in a NanoTemper Technologies MST machine (München, Germany). Data analysis was conducted using MO. Affinity Analysis v3.0.5 and Prism 9 software.

### MD simulations

The residues in the pocket interacting with acetyl-CoA were analyzed using PLIP (31). The Autodock Vina result was employed as the initial structure for simulations, and the ligand prepared by AmberTools21 (32). All simulations were run at 300 K using AMBER99SB force field with SCP216 solvent, and the system equilibrated to pH 7.0 using 150 mM NaCl in Gromacs-2022.4 (33,34).

Simulations were prepared by placing the starting structure in a cubic box that extended 1.2 nm beyond the protein in any dimension. The system was then energy minimized with the steepest descent algorithm until the maximum force below 1000 kJ/mol/nm using a cutoff distance of 1.2 nm for the neighbor list, van der Waals interactions and Coulomb interactions. For equilibrium run was conducted successively in NVT (constant-temperature, constant-volume) and NPT (constant-pressure, constant-temperature) ensemble, using V-rescale temperature coupling (time constant of 0.1 ps). The NPT ensemble in equilibrium using the isotropic pressure coupling with the Berendsen algorithm (time constant of 2 ps, 1 bar).

The production run was performed in the NPT ensemble at a pressure of 1 bar for a total of 1200 ns and repeat five times. Temperature and pressure were coupled using the V-rescale method (time constant of 0.1 ps) and isotropic pressure coupling with the Parrinello-Rahman algorithm (time constant of 2 ps), respectively.

### BATCH

Bacterial adenylate cyclase two-hybrid was used to test AccR dimerization (35). The coding sequences for AccR variants under test were cloned into pKT25 and pUT18C, respectively, and the resulting vector pair were co-transformed into *E. coli* BTH101. The strains having the vector pair were selected on LB plates containing 100 μg/ml ampicillin, 50 μg/ml kanamycin, 40 μg/ml X-gal and 0.5 mM IPTG.

### Other analyses

Student’s *t-*test was performed for pairwise comparisons. Data subjected to statistical analysis were presented as the mean of four biological replicates ± standard deviation (SD).

## Results

### Identification of SO_0839 as a regulator impacting growth

**Figure 2.**
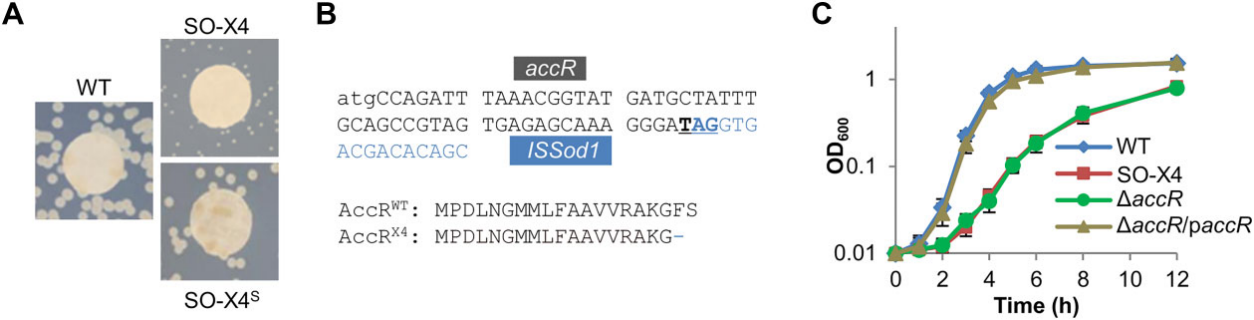
Identification of the mutation in spontaneous mutant SO-X4 of *S. oneidensis*. SO-X4 had a slow-growth phenotype. A whole-genome *S. oneidensis* expression library using pHG102 was introduced into SO-X4 and isolates that grew comparably to the WT were obtained. (**A**) Grown on LB agar plates. SO-X4^S^, the isolate that grew the same as WT. A paper disc of 8 mm was plated on the plates as a size reference. (**B**) The gene expressed in SO-X4^S^ turned to be *accR*. The nonsense mutation (T^55^TC→ T^55^AG, underlined) caused by an ISSod1 insertion was identified in the *accR* gene. AccR^X4^ represents the resultant protein. (**C**) Growth in LB. Fresh media were inoculated with overnight cultures by 200-fold dilution. p*accR*, a copy of the *accR* gene was expressed *in trans* for complementation under control of IPTG inducible promoter P*tac*, with 0.2 mM IPTG. Throughout this study, growth measurements were performed four times independently, and the data are presented as the mean ± SD (error bars).

This investigation began with the chance observation that an *S. oneidensis* spontaneous mutant SO-X4 displayed significantly retarded aerobic growth on LB agar plates and in broth compared to the wild-type (WT) (Figure 2A and B). To map the mutation, we constructed an *S. oneidensis* genomic library using the expression vector pHG102 driven by the *S. oneidensis arcA* promoter, whose activity is relatively constitutive (36,37). The library was introduced into SO-X4 to screen for genes capable of suppressing the growth defect, and several colonies of the WT size (SO-X4^S^) were obtained (Figure 2A). The plasmids recovered from these isolates all harbored SO_0839 (named as *accR* according to its function revealed in this study), which encodes an LTTR of 303 amino acids.

To confirm that *accR* is related to the growth defect, the *accR* region of SO-X4 was cloned and sequenced. There is an insertion of ISSod1 after nucleotide 55, resulting in a nonsense mutation (T^55^TC → T^55^GA) that changes the coding sequence to produce a protein of 18 amino acids in length (Figure 2B), which is unlikely to be functional. Then an *accR* in-frame deletion strain was constructed. This Δ*accR* strain was identical to SO-X4 with respect to growth in LB broth (Figure 2C). Similar growth defects were also observed with the defined medium (Supplementary Figure S2A), ruling out the possibility that the growth defect is associated with particular nutrients present in complex medium. Importantly, Δ*accR* could be successfully complemented with a copy of the *accR* gene expressed *in trans* (Figure 2C and Supplementary Figure S2A). Therefore, these data conclude that the loss of AccR results in the growth defect observed in SO-X4.

### AccR acts as an activator for *accS*

**Figure 3.**
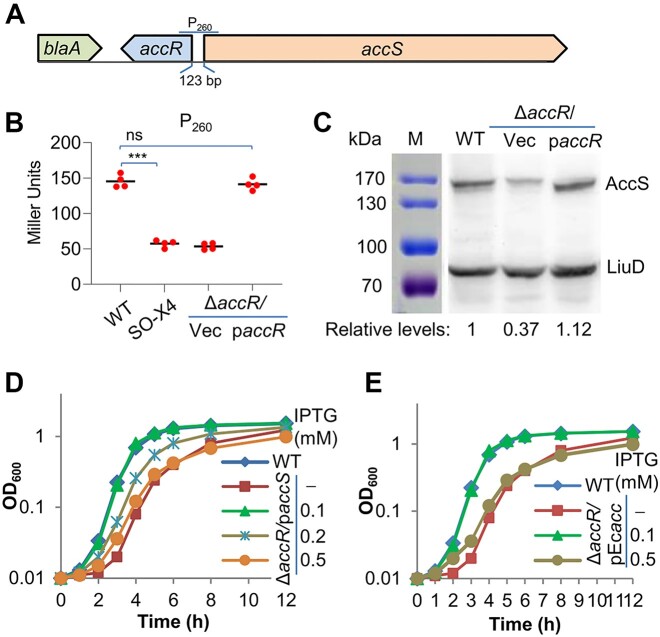
Reduced expression of AccS underlies growth defect of SO-X4. (**A**) The *accR* locus. The intergenic region is 123 bp in length. A fragment of 260 bp centered on the intergenic region (P_260_) was initially used for promoter activity assay. (**B**) Activity of P_260_ in cells grown in LB. The activity was assayed with an integrative *lacZ* reporter. p*accR*, a copy of the *accR* gene was expressed *in trans* for complementation under control of IPTG inducible promoter P*_tac_*, with 0.2 mM IPTG. Asterisks indicate statistically significant difference of the values compared (*n* = 4; ns, not significant; *P< 0.05, **P< 0.01, ***P< 0.001). (**C**) Endogenous biotinylated proteins identified by Western blot with the HRP-coupled anti-streptavidin antibody. In both (B) and (C), cells of strains under test grown to the mid-exponential phase (∼0.4 of OD_600_) were used. Complemented strains were grown with 0.2 mM IPTG. Shown are representative images from four independent experiments. (**D**) Effects of *S. oneidensis accS* expressed to varying levels on growth of Δ*accR*. (**E**) Effects of *E. coli acc* (*accA*, *accD* and *accBC* altogether) expressed to varying levels on growth of Δ*accR*.

The most likely explanation for the reduced growth phenotype resulting from the AccR loss would be altered expression of genes under AccR regulation. To identify the key targets, we first looked for genes in the proximity of *accR* as it is common that LTTRs and their targets form divergent operons (38,39). In *S. oneidensis*, the gene that is transcribed divergently from *accR* is *accS*, which encodes a multi-domain ACCase predicted before (18) (Figure 3A). This arrangement is conserved in *Shewanella* and is also found in some other genera of γ-proteobacteria, such as *Motilimonas*, *Parashewanella*, *Pseudomarivurvus* and *Exilibacterium*, to name a few (Supplementary Figure S1). The effect of the loss of AccR on expression of *accS* was then examined using an integrative *lacZ*-reporter, into which a DNA fragment of 260 bp centered on the *accR*/*accS* intergenic DNA sequence, which is 123 bp (Figure 3A), was introduced. Results showed that when AccR was depleted, expression of the *accS* gene decreased substantially, to only ∼35% of the WT level (Figure 3B). As ACCases are biotinylated in the BCCP subunit/domain, we also assessed changes in AccS abundance caused by the AccR loss with western blot using the HRP-coupled anti-streptavidin antibody. As reported before (40), two bands were detected: one is AccS migrating close to 170 kDa and the other is LiuD of ∼80 kDa, the biotin-binding subunit of methylcrotonyl-CoA carboxylase (694 amino acids) (Figure 3C). Apparently, the band corresponding to AccS was much weaker in Δ*accR* compared with that in the WT, and band intensity quantification by ImageJ supported the results of the *lacZ*-reporter. As an LTTR, it was possible that AccR may self-regulate its own transcription. However, this does not seem to be the case, because the absence or overproduction of AccR did not change the activity of the *accR* promoter compared to that observed in the WT (Supplementary Figure S2B).

To further confirm that AccR regulates *accS* transcription, we introduced into Δ*accR* a copy of *accS* under the control of IPTG-inducible promoter P*_tac_*, whose activity has been shown to increase progressively with concentrations of IPTG up to 1 mM (21). The growth defect resulting from the AccR loss was fully recovered with 0.1 mM IPTG (Figure 3D and Supplementary Figure S2C), supporting that the reduced AccS production underlies the growth defect. Intriguingly, when IPTG levels were further augmented to 0.2 mM or more, growth of Δ*accR* became impaired (Figure 3D; Supplementary Figure S2C and D). A similar scenario was also observed from the WT strain overexpressing the gene, indicating that AccS in excess is detrimental (Supplementary Figure S2E). Furthermore, we cloned the *E. coli accA, accD* and *accBC* genes together into the same vector and expressed them in Δ*accR*. In the presence of 0.1 mM IPTG, growth of the Δ*accR* strain became indistinguishable from that of the WT (Figure 3E). Consistently, when overproduced, *E. coli* ACCase also caused growth inhibition (Figure 3E). These data, altogether, manifest that the growth defect of Δ*accR* is due to the compromised AccS production, and AccR functions as a transcriptional activator for the *accS* gene.

### AccR functions as a conventional LTTR in regulating *accS* transcription

**Figure 4.**
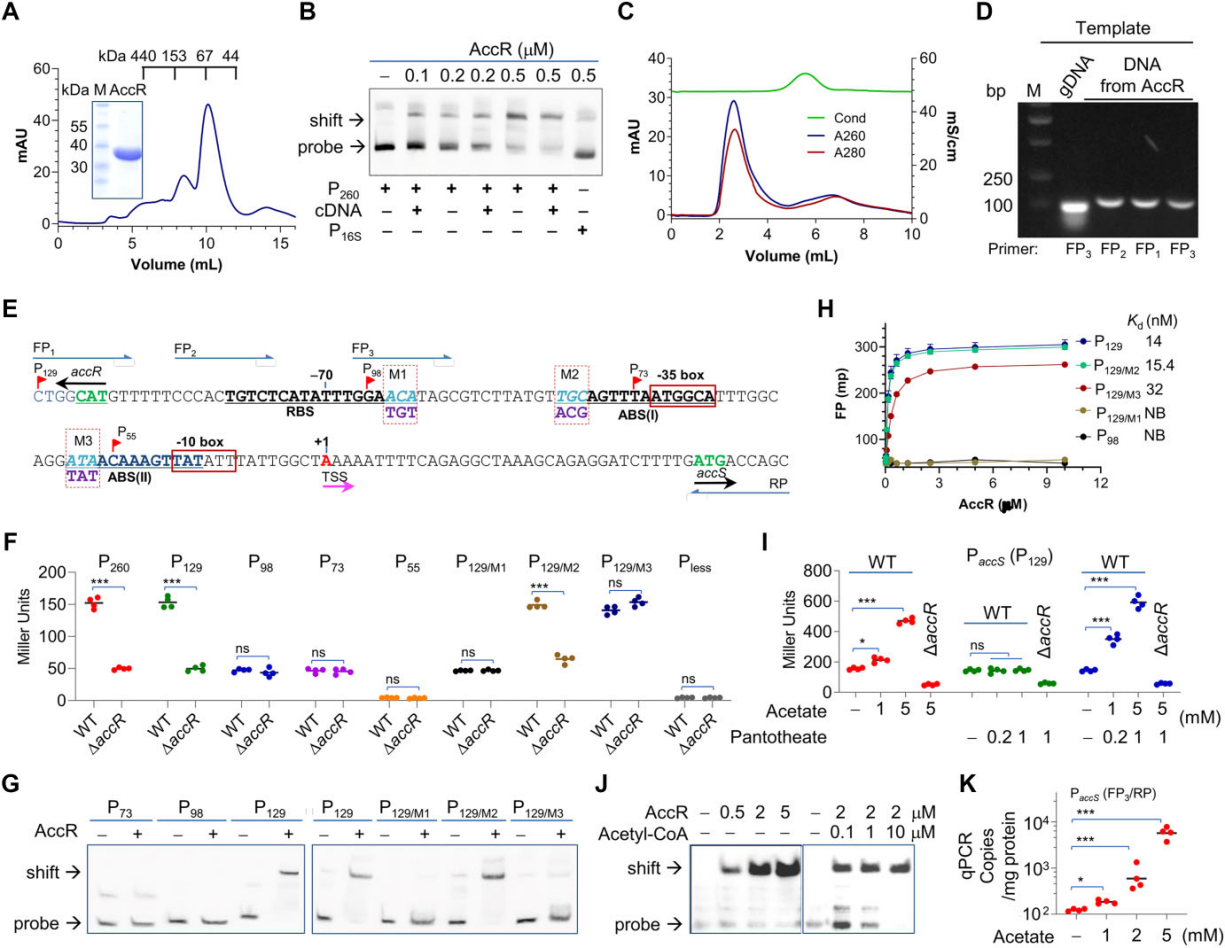
AccR regulates *accS* transcription in response to acetyl-CoA. (**A**) Gel exclusion chromatographic profile of His-tagged recombinant AccR purified from *E. coli* run on a Superdex 200HR 10/30 column. The expected peak was eluted at the position of 10.3 ml. The inset gel is SDS-PAGE result. Molecular weight of recombinant AccR is about 36 kDa. M, marker. (**B**) *In vitro* interaction of His-tagged AccR and the *accR*/*accS* promoter sequence (P_260_) revealed by using EMSA. The digoxigenin-labeled DNA probes were prepared by PCR. The EMSA was performed with 1 μM probes and various amounts of proteins as indicated. Nonspecific competitor DNA (cDNA, 2 μM poly(dIdC)) was included. The 16s rRNA gene promoter was used as control promoter (P_16S_). (**C**) His-tagged recombinant AccR purified from *S. oneidensis* contains DNAs. Shown are purified proteins after a desalting column. A280 and A260 represent protein and DNA, respectively, and Cond, conductivity of the sample. (**D**) Identification of DNA fragments bound to AccR shown in (C). DNA extracted was used as the template for PCR amplification with primers RP and one of FP_1_, FP_2_, and FP_3_ shown in (**E**). (E) The *accS* promoter region. Red arrows, DNA fragments subjected to promoter activity assays; for example, P_129_, from -129 to translation start site ATG. M1, M2 and M3, mutations. (**F**) Activity of the promoter region sequences. P_less_, the promoter-less control. (**G**) EMSA analysis of binding of AccR to the *accS* promoter region sequences. (**H**) DNA-binding of AccR assessed by FP. Data are represented by the mean ± SD (error bar) of at least three independent experimental measurements and were averaged for calculating the binding affinity (*K*_d_). (**I**) Effects of exogenous acetate and pantotheate on *accS* expression in WT and Δ*accR* by measuring P*_accS_* activity. Cells were collected 30 min after the addition for β-galactosidase assay. (I) EMSA analysis of effect of acetyl-CoA addition on binding of AccR to P*_accS_* (P_129_). (**J**) qPCR analysis of effect of acetate addition on binding of AccR to P*_accS_* (P_129_). DNA extracted from cells grown to the mid-exponential phase supplemented with acetate was quantified with primers RP and FP_3_. In (A–D), (G) and (J), representative images represent three independent experiments. In (F), (H), (I) and (K), asterisks indicate statistically significant difference of the values compared (*n* = 4; ns, not significant; *P< 0.05, **P< 0.01, ***P< 0.001).

We then tested whether AccR interacts with the promoter region of the *accS* gene. To this end, the recombinant AccR (35.6 kDa calculated molecular weight) with a His_6_-tag at the C-terminus was expressed in *E. coli* and purified to near homogeneity (Figure 4A). The His-tagged AccR was found to be functional *in vivo* as its expression could fully complement the growth defect of the Δ*accR* strain (Supplementary Figure S3A). On the SDS-PAGE, the recombinant protein migrated to a position consistent with its molecular weight, but the FPLC profile showed two eluted peaks with estimated molecular weights of ∼67 and ∼140 kDa, indicating that AccR could exist as a dimer and a tetramer in solution (Figure 4A). This is not surprising as LTTRs generally form dimers and tetramers (38,39). EMSA assay was then performed with purified AccR of varying amounts and the intergenic DNA fragment used above in promoter activity assay. As shown in Figure 4B, AccR effectively interacted with this DNA fragment but not the control promoter sequence from the 16s rRNA gene, supporting that AccR regulates expression of the *accS* gene in a direct manner.

The recombinant His_6_-tagged AccR proteins were also expressed and purified from the *S. oneidensis* Δ*accR* strain. In contrast to those purified from *E. coli*, these proteins were found to be bound to DNA sequences (Figure 4C). DNAs from the protein samples were then isolated and used as the template for PCR amplification with primers targeting the *accR*/*accS* intergenic region. With three different forward primers (FP_1_, FP_2_ and FP_3_) and one reverse primer (RP) overlapping the translation start codon (**A**TG), three fragments of 130, 120 and 110 bp were successfully amplified (Figure 4D and E), indicating that the bound DNA fragment covers the promoter region of *accS*. Subsequently, the purified protein samples were treated with DNase and subjected to sequencing for pinpointing the sequence protected by AccR. Most of the sequences were found to cover the region from -110 to -40 (Figure 4E), which is in excellent agreement with the results of *in vitro* DNA footprinting assay (Supplementary Figure S3B). Given that the transcription start site (TSS) of the *accS* gene was determined to be an adenosine (+1) located at 35 bp upstream of ATG by 5′-RACE assays (Figure 4E), it is clear that AccR interacts with the DNA sequence immediately upstream of the TSS.

To regulate a divergent promoter, LTTRs generally bind to DNA sequences consisting of three different functional palindromic subsites (LTTR binding motifs): a recognition binding site (RBS) and two activation binding sites ABS(I) and ABS(II), which are often found near positions -65, -35 and -10 relative to the TSS, respectively (41,42). We then examined the AccR-binding region and three palindromic sequences were found: LTTR-binding motifs RBS (-79 − -62), ABS(I) (-48 − -33) and ABS(II) (-24 − -11) (Figure 4E). However, only RBS appears to be conserved compared to those established for LTTRs that control a divergent promoter (Supplementary Figure S3C).

By using the *lacZ*-reporter, activities of multiple *accS* promoter variants were assayed. These variants contain sequences covering the entire or partial intergenic region, which are 129, 98, 73 and 55 bp long, named P_129_, P_98_, P_73_ and P_55_, respectively (Figure 4E). Apparently, the 129-bp fragment P_129_ exhibited activity comparable to the 260 bp fragment (P_260_) used above for the DNA-binding assay, indicating that P_129_ is sufficiently long to cover all binding requirements for full activity (Figure 4F). In contrast, P_55_ was inactive, not significantly different from the promoter-less (P_less_) control (Figure 4F), which is not surprising because this fragment lacks an essential element for activity, the -35 box. While both P_98_ and P_73_ had activity comparable to P_129_ in the absence of AccR, they were not responsive to AccR (Figure 4F), implying that AccR may not be able to interact with them. Our data thus suggest that the RBS is essential for the activation of AccR, and the loss of either ABS(I) or ABS(II) renders the promoter to be AccR-independent.

*In vitro* binding assays were further performed to confirm the direct binding of AccR to its regulatory promoter sequences. P_129_ mutants M1, M2 and M3, which have a disrupted RBS, ABS(I) and ABS(II) site, respectively, were constructed and referred to as P_129/M1_, P_129/M2_ and P_129/M3_ (Figure 4E). While the mutation within RBS rendered P_129/M1_ to be independent of AccR, P_129/M2_ behaved like the WT P_129_ in the *lacZ*-reporter essay (Figure 4F). Consistently, EMSA results using synthetic oligos indicated that RBS is crucial for AccR binding whereas ABS(I) appeared to have a minimal effect (Figure 4E). Intriguingly, the P_129/M3_ promoter exhibited high constitutive activities comparable to the activated state both in presence and absence of AccR, while the binding between the protein and the DNA was substantially compromised (Figure 4F and G). Moreover, we measured the binding affinity of AccR for these regulatory promotor sequences using FP assays. Results from these FP experiments showed comparable binding affinity of AccR for P_129_ and P_129/M2_, >2-fold reduction in binding affinity for P_129/M3_ and no binding for P_98_ or P_129/M1_ (Figure 4H). These data overall suggest that AccR regulates transcription of the *accS* gene in a way similar to the established model for LTTRs that control a divergent promoter.

Moreover, given that acetyl-CoA is the substrate of AccS, we hypothesized that acetyl-CoA may be the effector of AccR. To test this *in vivo*, we assessed activity of AccR by monitoring P*_accS_* (P_129_, from this point forward unless otherwise noted) activity in the WT and Δ*accR* cells grown with addition of acetate, which is commonly used to efficiently increase acetyl-CoA production in bacteria (43). In the WT, P*_accS_* (P_129_) activity was significantly enhanced by the addition, ∼1.9-fold with 5 mM acetate (Figure 4I). Moreover, we further assessed the effect of pantotheate, a precursor of CoA that can be easily taken up into the cell, in the absence and presence of acetate on *accS* expression. While the addition of pantotheate alone up to 1 mM had no detectable impact on P*_accS_* activity in either WT or Δ*accR*, it enhanced *accS* expression further when combined with acetate (Figure 4I). This effect appeared particularly significant with acetate at low concentration (1 mM). In contrast, P*_accS_* activity in the Δ*accR* strain was not affected significantly by addition of either, supporting that AccR is responsive to intracellular levels of acetyl-CoA.

Subsequently, we examined whether acetyl-CoA promotes binding of AccR to P*_accS_* with EMSA. Unexpectedly, the difference in binding between the absence and presence of acetyl-CoA was not significant (Figure 4J). Similar results were observed with malonyl-CoA (Supplementary Figure S3D). We then took advantage of the finding that the *accS* promoter fragments are complexed with purified recombinant AccR proteins, which allows us to quantify DNA bound from AccR proteins within *S. oneidensis* Δ*accR* cells grown in LB supplemented with acetate. By using primers FP_3_ and RP in qPCR (Figure 4E), we found that copies of the fragment increased with acetate concentrations (Figure 4K), supporting that acetyl-CoA indeed improves interaction between AccR and P*_accS_ in vivo*. These data, all together, suggest that regulation of AccR is responsive to acetyl-CoA but additional factors may be required for interaction of AccR with acetyl-CoA.

### Crystal structure of the AccR EBD

**Figure 5.**
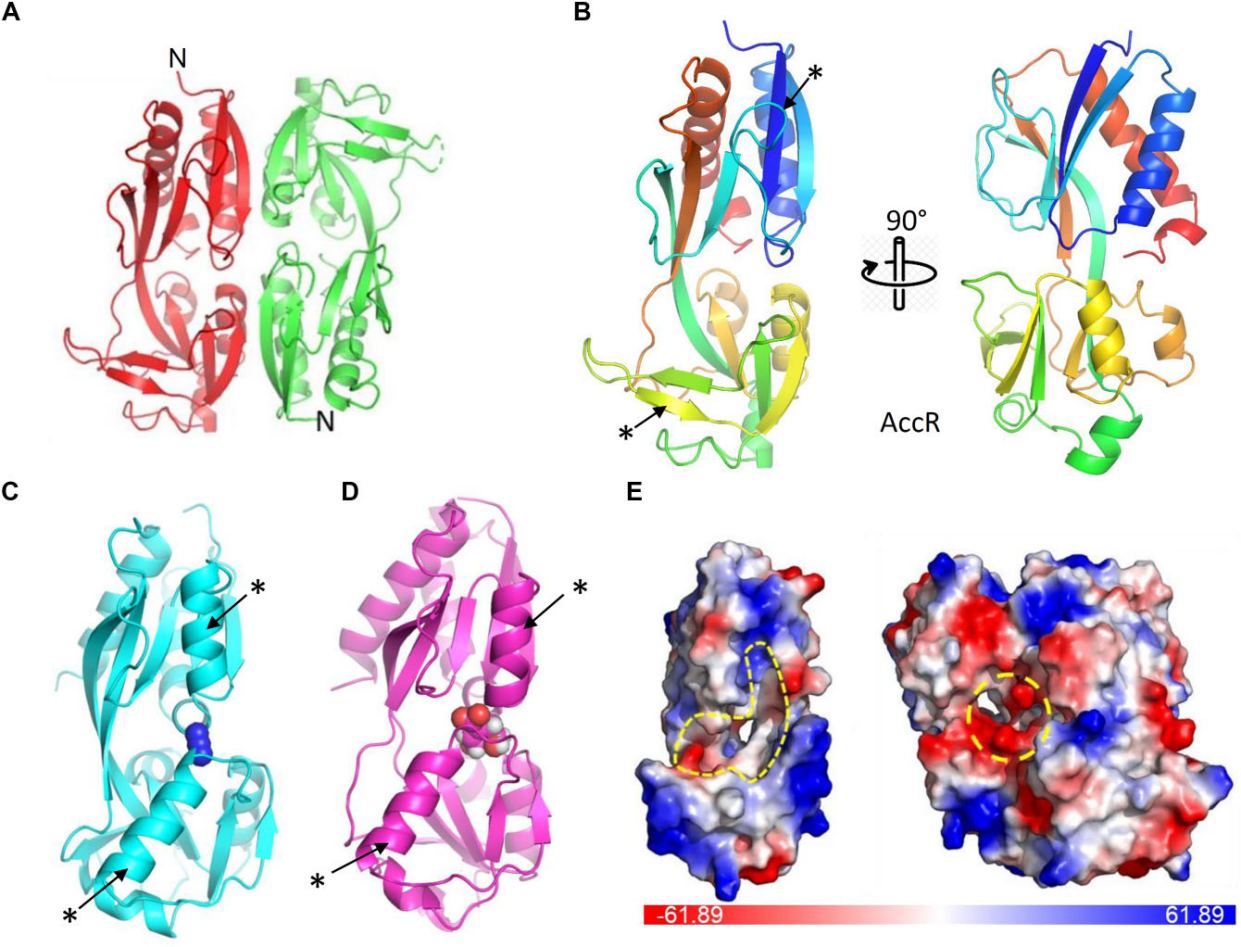
Crystal structure of the AccR EBD. (**A**) An AccR EBD dimer. The two subunits are colored differently in red and green. The molecule is viewed along the 2-fold symmetry axis. (**B–D**) Ribbon diagram of the EBD from AccR, CynR and QuiR. The bound ligands in CynR and QuiR are shown by spheres. Stars and arrows highlight the two major structural differences between AccR and CynR/QuiR. (**D**and**E**) Surface representation of EBD monomer and dimer. The molecule is colored by electrostatic potential with positively and negatively charged surfaces shown in blue and red, respectively. The putative ligand binding pocket is outlined.

Like other LTTRs, AccR is predicted to form a N-terminal winged helix-turn-helix (wHTH) DNA-binding domain (DBD) and a C-terminal EBD (also called regulatory domain, RD) that are connected by a linker made of an extended helix and a flexible hinge (Supplementary Figure S4A) (44). Although the structure of AccR predicted by AlphaFold2 (AFDB ID: AF-Q8EIK0-F1) exhibits typical features of LTTR regulators (Supplementary Figure S4B), it has an average pLDDT value of 81.31 for the whole protein and <50 for some surface elements, the lowest among LTTR regulators in *S. oneidensis*, suggesting relatively low confidence in accuracy. Therefore, attempts were made to determine the crystal structures of AccR. Despite numerous trials, we were not able to obtain any crystal hits for the full-length AccR. Nevertheless, the AccR EBD (residues 86–303) was successfully crystallized and its structure solved at 1.4 Å resolution (PDB ID: 9BCE) (Figure 5A). EBD crystals were rod-shaped, belonging to the space group C2. An EBD dimer was found in each asymmetric unit, with the two subunits arranged in an antiparallel manner (Figure 5A). The final structural model of AccR EBD contains residues 89–302 for subunit A and residues 90–300 for the subunit B, except for residues 132–136 and 206–207 in two surface loops of the subunit B that are structurally disordered. Superimposition of the two EBD subunits in a dimer gave an overall r.m.s.d. of 0.25 Å for 204 common C_α_ atoms, suggesting nearly identical conformation.

Each AccR EBD can be further divided into two subdomains of mixed α/β folds, EBD-1 (residues 89–161 and 264–303) and EBD-2 (residues 162–263), like in other LTTRs such as LeuO-EBD (PDB ID: 6GZ0) (45). While EBD-1 consists of strands β3–6 and β12 and helices α5, α9–10, EBD-2 is composed of strands β6–12 and helices α6–8 (Figure 5B). EBD-1 and EBD-2 are connected by two extended, antiparallel cross-over strands β6 and β12. Compared to the predicted structure, some differences in secondary structures were observed. Apparently, our structure contains long-coil regions, where the AI-generated counterpart predicts short helices to exist (Supplementary Figure S4A–E). The relative spatial orientation of ED1 and EB2 is further supported by extensive noncovalent interactions across the two subdomains as well as interactions with the others subunit (see more detailed discussion below). No extra electron densities were found in the putative ligand-binding site located between EBD1 and EBD2, however, indicating crystallization of the apo from of the protein

A Dali search against the RCSB Protein Data Bank returned >30 transcriptional regulators and other structural homologs, with the three top hits being CrgA from *Neisseria meningitidis* (PDB ID: 3HHG, *Z* score = 17.1, with >2 being significant), AphB from *Vibrio vulificus* (PDB ID: 5FHK, *Z* score = 16.5) and HypT from *Salmonella enterica* (PDB ID: 5YDO, *Z* score = 15.9) (46–48). Similar EBD dimers were also observed in these and other reported LTTR structures of both full-length and the EBD domain only (39,45–48). In such anti-parallel dimers, the two N-terminal DBDs from the two subunits would point toward opposite directions (Figure 5A; Supplementary Figure S5A and B). It has been proposed that LTTRs would oligomerize into high-order assemblies such a tetramer and even octamer, introducing DNA looping during DNA binding (39). In the AccR EBD dimer, ∼3200 Å^2^ of surface area is buried at the dimer interface, which is mostly mediated by α5, β4 with α7 and β10 from the adjacent subunit (Figure 5A), suggesting stable molecular interactions.

To gain insights into the ligand binding activity of AccR, we superimposed the structure of AccR EBD with two other closely related LTTR EBD structures with bound ligands: *E. coli* CynR with its specific effector azide (PDB ID: 3HFU, *Z* score = 15.2) and the EBD of QuiR from *Listeria monocytogenes* bound with shikimate (PDB ID: 5TED, *Z* score = 14.8) (Figure 5C and D) (49,50). Structural comparison indicates that AccR EBD has a large, ‘primary’ funnel-shaped ligand binding pocket within a monomer (Figure 5B and E). This funnel-shaped pocket, which runs through the entire molecule, is 25 Å by 20 Å wide near its opening, with most of the surface being neutral to positively charged. The ligand binding pocket is significantly smaller in CynR and QuiR because of two major structural differences: (i) the loop connecting β4 and β5 in AccR is occupied by a bulky α-helix in both CynR an QuiR, and (ii) the hairpin β4-β5 in AccR is replaced by another extended α-helix in CynR and QuiR (Figure 5B–D). In addition, another ‘secondary’ pocket is found at the regulatory domain dimerization interface and contains S103 and E105 from both chains of the dimer (Figure 5E and Supplementary Figure S5C). A similar secondary pocket is also present in AphB of *Vibrio*, which has been shown to bind the small molecule BP-15 although its natural ligand is yet to be identified (Supplementary Figure S5D) (47,51). However, this secondary pocket found in AccR may not interact with acetyl-CoA because it is a negatively charged cavity as seen by the surface charge representation (Figure 5E), and acetyl-CoA is also negatively charged.

### Acetyl-CoA interacts with AccR but additional effector is required for full activity

**Figure 6.**
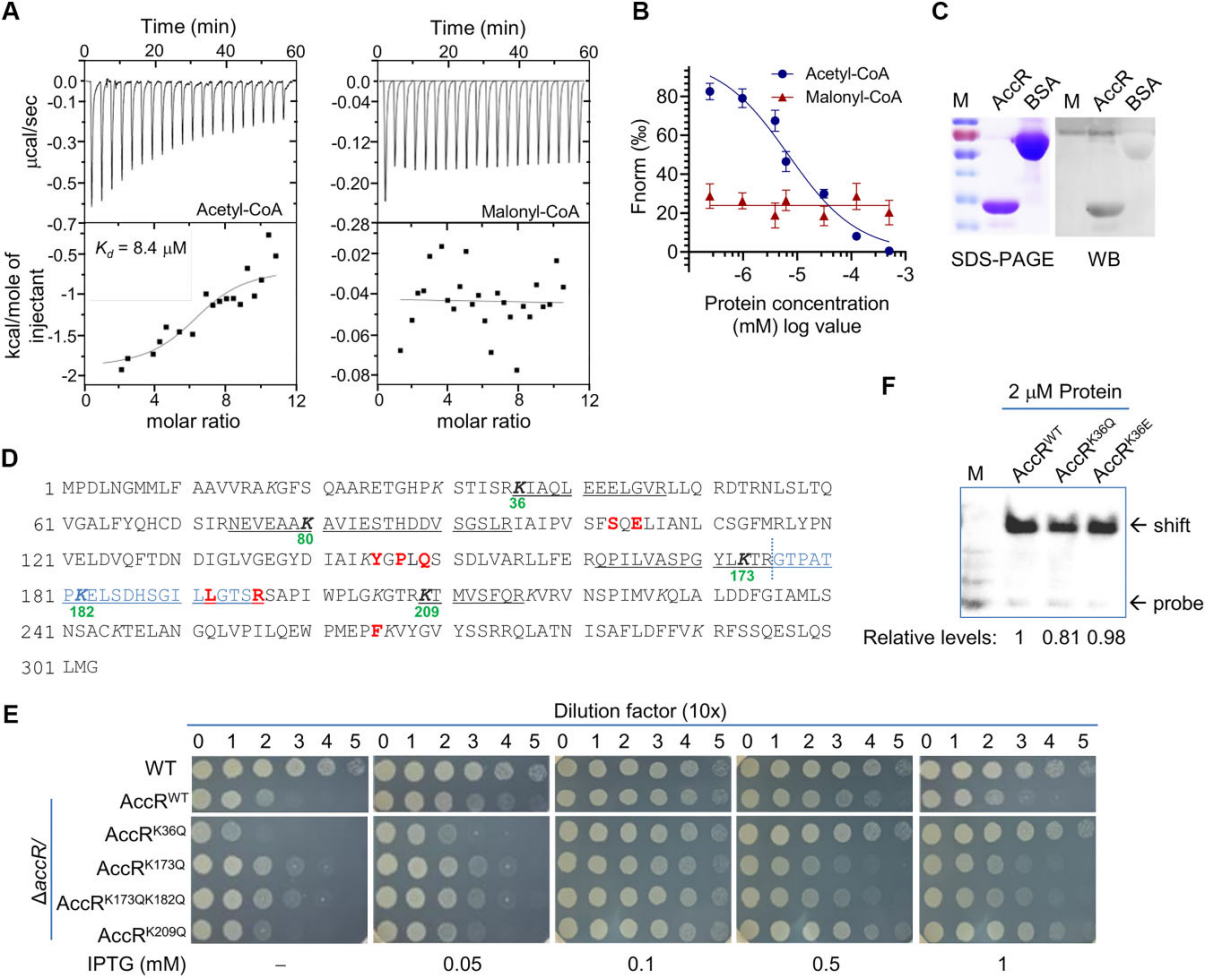
Acetyl-CoA is one of the effectors for AccR. (**A**) Thermophoretic analysis of the interaction of AccR with acetyl-CoA or malonyl-CoA. The ITC binding isotherm of acetyl-CoA or malonyl-CoA (1.8 mM) titrated into the AccR EBD (36 μM) at 298 K in HEPES buffer, represented as the heat change (cal/s) upon injection over time. Shown are representative images from three independent experiments. (**B**) Normalized fluorescence changes were measured to yield binding curves. Normalized fluorescence is defined as Fhot/Fcold (refer to Supplementary Figure S6B). Each datum is the mean ± standard error of the mean (SEM) of at least three independent experiments using the same batch of purified protein. (**C**) AccR is acetylated. WB of AccR with acetylated-lysine antibody. BSA was used as control. (**D**) AccR amino acid sequence coverage obtained from LC-MS/MS analysis. The peptides underlined contain acetylated lysine residues, whose positions are given. Two acetylated peptides next to each other are in black and blue. Approximately 89% of the AccR amino acid sequence has been mapped in the MS study. Residues in red, which are proposed to be important for maintaining the effector-binding pockets, are subjected to site-directed mutational analysis (also refer to Figure 7A and Supplementary Figure S5C). (**E**) Growth of Δ*accR* expressing one of AccR variants to varying levels on LB agar plates. Acetylation residues were mutated individually and in combination (also refer to Supplementary Figure S7A and D). (**F**) EMSA analysis of effect of indicated mutations on binding of AccR to P*_accS_* (P_129_). Panels (A), (C), (E) and (F) show representative data or images from three independent experiments. In (B), data are represented by the mean ± SD (error bar) of at least three independent experimental measurements.

The data presented above suggest that acetyl-CoA is likely one of the effectors for AccR. To substantiate the direct interaction between the regulator and acetyl-CoA, we purified His_6_-tagged full-length AccR and the EBD and tested their ability to bind acetyl-CoA by using ITC and MST. Unfortunately, both approaches could not be carried out with full-length AccR because it quickly aggregated and precipitated after the addition of acetyl-CoA. With the EBD, we managed to observe the binding of AccR to acetyl-CoA but not malonyl-CoA in the ITC assays that were carried out in a buffer of a low enthalpy of ionization (HEPES) (Figure 6A). However, acetyl-CoA affinity of the AccR EBD is rather high (*K*_d_ = 8.4 ± 1.3 μM) and the binding could not be observed in a Tris buffer (Supplementary Figure S6A), which has a high enthalpy of ionization, implying that the binding of acetyl-CoA to AccR may be weak and/or unstable. In the MST assays, a comparison of the AccR EBD interacting with acetyl-CoA and malonyl-CoA supported that the protein could only interact with acetyl-CoA (Figure 6B and Supplementary Figure S6B). The binding affinity determined by MST was 7.3 ± 1.1 μM, and notably, the binding with proteins at low concentrations (≤2.56 μM) was found to responsive to acetyl-CoA, but not when the concentration was over 5 μM (Supplementary Figure S6B). These data, collectively, suggest that although acetyl-CoA interacts with AccR directly, the binding is probably unstable and additional effecting molecule is likely required for its regulation activity.

We then examined whether protein acetylation plays a role in activation of AccR, a posttranslational modification (PTM) conserved in all domains of life that acetyl-CoA could cause (52). In bacteria, the most common form of acetylation, transferring the acetyl moiety of acetyl-CoA to *N*^ϵ^-amino groups of lysyl residues of a protein, is catalyzed by homologs of the yeast Gcn5 histone *N*-acetyltransferase (GNAT), and can be reversed by deacetylases (53). His_6_-tagged AccR, which is functional (Supplementary Figure S3A), was purified from Δ*accR* cells growing normally and subjected to an antiacetyl-lysine Western blot analysis. Apparently, the result demonstrated that AccR was acetylated (Figure 6C). By employing high-resolution mass spectrometry, 5 out of 14 lysine residues of AccR were found to be acetylated, including K36, K80, K173, K182 and K209 (Figure 6D).

Impacts of acetylation at these residues on the activity of AccR, alone and in combination, were then assessed with AccR variants carrying K to Q point mutations. By monitoring complementing effects of these AccR variants, we found that the K36Q mutation is only one that was unable to fully correct the growth defect caused by the loss of AccR (Figure 6E and Supplementary Figure S7A). Given that this residue is in the DNA-binding region, we reasoned that the mutation may compromise DNA-binding, thereby regulatory activity of AccR. This notion was tested with EMSA with AccR^WT^, AccR^K36Q^, as well as AccR^K36E^, which mimics a constitutively acetylated lysine residue in many scenarios (52). Indeed, AccR^K36E^ behaved the same as AccR^WT^ (Supplementary Figure S7A). EMSA analysis of these three recombinant AccR variants, which were purified from *E. coli* (Supplementary Figure S7B), revealed that AccR^K36Q^ displayed a modestly reduced binding capacity to P*_accS_* (Figure 6F). In addition, P*_accS_* fragments bound to these variants *in vivo* were quantified with qPCR. Consistently, the DNA bound to AccR^K36Q^ were found to significantly less than those bound to AccR^WT^ or AccR^K36E^ (Supplementary Figure S7C). On the contrary, the mutations in the EBD domain, even in combination, did not show detectable impact on growth (Supplementary Figure S7D).

Like *E. coli*, the *S. oneidensis* genome encodes a large number of GNATs (i.e. at least 32) but only one deacetylase, CobB (SO_1938) (52). To provide additional evidence to support that the regulatory function of AccR is independent of acetylation, a *cobB* deletion mutant was constructed, which grew comparably relative to the WT (Supplementary Figure S7A). Overexpression of this gene also had no detectable impact on growth (Supplementary Figure S7A). Consistent with its function, we found that the loss of CobB resulted in increased abundance of acetylated AccR (Supplementary Figure S7E). However, CobB either in its absence and overproduction had no detectable effect on AccS abundance (Supplementary Figure S7F). Altogether, these data ruled out the possibility that AccR is activated via acetylation.

### Molecular dynamics simulations for acetyl-CoA and AccR complexation

**Figure 7.**
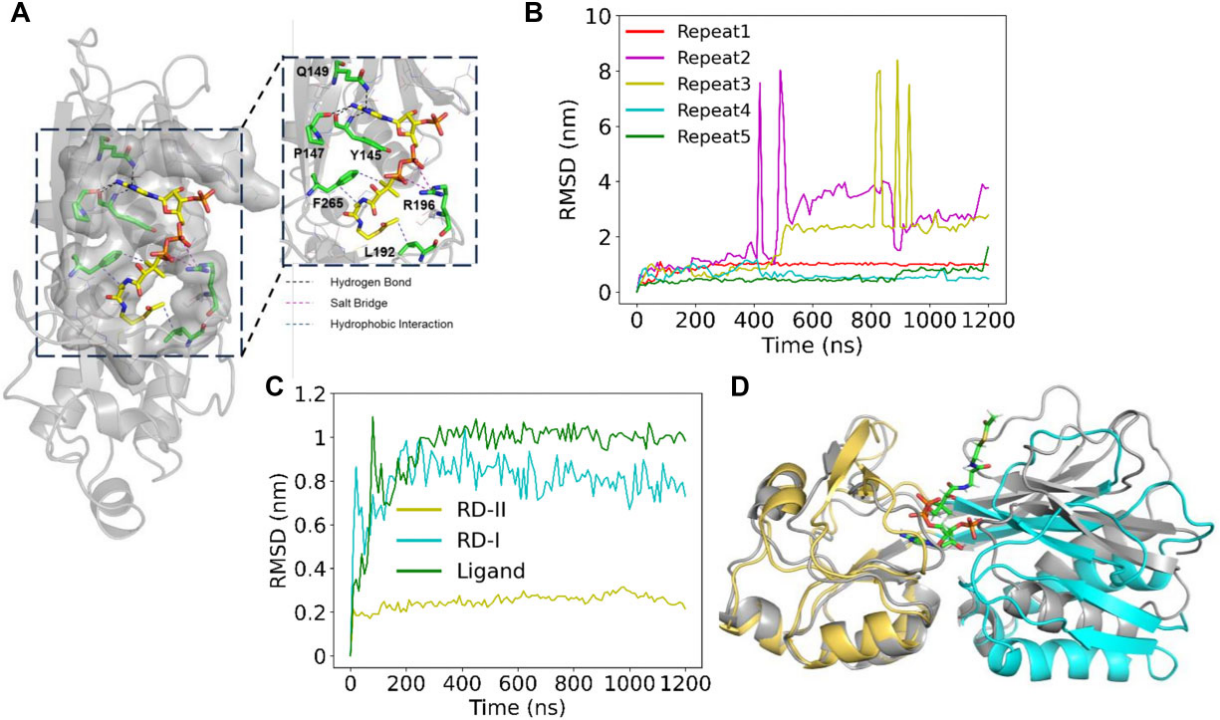
MD simulations of AccR interacting with acetyl-CoA. (**A**) The regulatory domain of AccR is depicted in cartoon form, and the ligand is represented as sticks with C atoms in yellow. Residues within 0.5 nm of the ligand are displayed as lines and transparent surfaces. The residues interacting with the ligand are highlighted in sticks with C atoms colored green. In the enlarged view, acetyl-CoA is situated in the pocket and is in contact with six residues; the interactions between atoms are denoted by different colored dashes. (**B**) RMSD of the ligand in five simulations. Acetyl-CoA in repeats 1 and 4 is relatively stable, but their RMSD values differ, indicating different ligand poses. In repeats 2 and 3, acetyl-CoA escaped from the pocket after ∼400 ns and, in repeat 5, escaped near the end. (**C**) Comparison of the RMSD of two regions and acetyl-CoA in repeat 1, revealing that RD-I’s RMSD tends to stabilize along with the stability of the ligand. (**D**) The initial (gray) and final (RD-I in cyan and RD-II in yellow) regulatory domains in repeat 1′s trajectory were compared. The two structures were aligned based on residues 162–187 and 222–264 in RD-II. The structure of RD-I has undergone significant changes. The acetyl-CoA in the final regulatory domain is depicted as sticks.

To address the unstable interaction between acetyl-CoA and AccR as measured by ITC, we further analyzed the pocket for effectors between RD-I and RD-II using CASTp (54) and SwissDock (55) (Supplementary Figure S8A and B). The pocket identified by both programs is in line with that present in each monomer as shown in Figure 5E. Subsequently, acetyl-CoA was docked to the pocket using AutoDock Vina (56). The most favorable pose was selected based on scoring and clustering for further analysis and MD simulations. AccR primarily binds acetyl-CoA through the pocket residues Y145, P147, Q149 from RD-I, and F265, L192, R196 from RD-II. Specifically, Y145, P147 and Q149 form hydrogen bonds with the N atoms of the adenine moiety of acetyl-CoA. R196 forms salt bridges with two consecutive phosphate groups, while L192 and F265 engage in hydrophobic interactions with the acetyl and coenzyme A moieties, respectively (Figure 7A).

To further illustrate the pocket’s affinity for acetyl-CoA, we conducted five independent 1200 ns MD simulations (named repeat 1–5) for the ligand and protein solution system. While in repeat 1 the RMSD of the ligand is very stable after 300 ns, in other repeats acetyl-CoA was found to be unable to stably bind to the pocket, falling off from the protein at different times (Figure 7B). Further analysis of repeat 1 revealed that RD-I tends to stabilize along with the stability of the ligand (Figure 7C) and RD-I undergoes an overall twist relative to RD-II after binding the ligand through structural comparison (Figure 7D). Overall, these data support that acetyl-CoA is able to interact with AccR, and by doing so the molecule has an inducible fit effect on the regulator, but to form the stable complex additional factors are required.

### Both putative effector-binding pockets are functionally important

**Figure 8.**
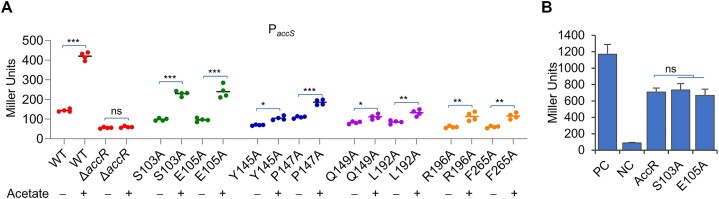
Both putative effector-binding pockets are functionally important. (**A**) Alanine scanning was carried out on residues predicted to be involved in formation of two effector-binding pockets. In Δ*accR*, each AccR variant was expressed with 0.2 mM IPTG and expression of *accS* was measured. The experiment was carried out the same as in Figure 2B w/o 5 mM acetate (refer to Supplementary Figure S9A and B for results of more residues). (**B**) Bacterial two-hybrid (BATCH) assay for detecting the interaction between two monomers. The interaction would activate expression of *lacZ* genes, which can be detected by β-galactosidase activity assay. The activities of β-galactosidase in BTH101 reporter strains carrying two vectors expressing the same AccR variants grown on LB plate containing 40 μg/ml X-gal and 0.5 mM IPTG at 30°C for 24 h were measured and presented as the mean of four replicates ± SD (error bars) in Miller Units.

To provide additional evidence to support that acetyl-CoA is an effecting molecule for AccR, we generated and characterized an array of AccR variants, with residues predicted to be crucial to effector-binding pockets subjected to alanine scan (Figures 6B and 7A; Supplementary Figure S5C). All of the AccR variants were expressed in the Δ*accR* strain with 0.2 mM IPTG and their impacts on *accS* expression were assessed. At this level of induction, while AccR^WT^ fully restores WT-level expression of *accS* in the Δ*accR* strain (Figure 2B), several variants showed heavily compromised activity (Figure 8A and Supplementary Figure S9A). These included AccR^S103A^, AccR^E105A^, AccR^Y145A^, AccR^P147A^, AccR^Q149A^, AccR^L192A^, AccR^R196A^ and AccR^F265A^. The residues in the first two variants are key to the pocket located at the interface of the dimer whereas the residues in the rest form the pocket present in the monomer (Figure 6B and Supplementary Figure S5C). In contrast, alanine replacement of the residues not involved in formation of the predicted acetyl-CoA binding pocket did not significantly affect regulation (Supplementary Figure S9A), suggesting that both pockets are important for regulatory activity. Reduced activities from these variants were also observed in the presence of 5 mM acetate (Supplementary Figure S9B). Importantly, the Δ*accR* strain expressing one of the AccR variants with residues predicted to be critical to the monomer pocket appeared to be substantially impaired in response to acetate addition (Figure 8A), supporting that these residues may interact with acetyl-CoA. In contrast, with AccR^S103A^ or AccR^E105A^, the Δ*accR* strain became responsive to the acetate addition (Figure 8A), suggesting that these two mutations do not affect the interaction between the protein and acetyl-CoA. Moreover, by using bacterial two-hybrid system (BATCH), we validated that neither AccR^S103A^ nor AccR^E105A^ significantly affected interaction between two monomers (Figure 8B), implying that the dimerization is not accountable for the reduced regulatory activity of these two variants. Overall, these data are in accord with the proposal that acetyl-CoA acts as an effector for AccR, and additional ligands are required. Nevertheless, other possibilities, such as the ligand may be a molecule derived from acetyl-CoA that fits the pocket present in the monomer, could not be completely ruled out.

## Discussion

FAS is not only an essential metabolic process for virtually all living organisms but also serves as an important precursor source for secondary metabolite generation. As ACCase catalyzes the first committed step of the biosynthesis, regulation of this enzyme, in terms of both quantity and activity, critically determines cellular lipid homeostasis. However, although studies into activity regulation have been many (2), prior to this study, transcriptional regulators that specifically regulate expression of *acc* genes have not been described. Here, we report identification and characterization of AccR, an LTTR that activates *accS* expression in response to cellular acetyl-CoA levels.

Identification of this regulator stems from a spontaneous mutant that carries a severe growth defect under normal growth conditions. The phenotype is caused by an ISSod1 insertion into the *accR* coding region, which leads to production of a substantially truncated protein that cannot be active. Intriguingly, the consequence of this mutation is in contrast to those of the same ISSod1 insertions reported before in *S. oneidensis*, which always generate a constitutive hybrid promoter, resulting in forced expression of the gene that follows (57–59). Thus, this type of DNA translocation has a more profound impact on genome evolution than expected previously.

Bacteria within the *Shewanella* genus are ubiquitously distributed in global water bodies and play an important role in sustaining and preserving aquatic ecosystems because of their unparallel physiological and respiratory versatility (60,61). Intriguingly, in these microorganisms FAS and its regulation has been found to carry many novel features, largely based on intensive studies of genus representative *S. oneidensis* (21,62–64). Unlike most of ACCases found in bacteria, the *S. oneidensis* counterpart is a single polypeptide composed of multiple functional domains as found in eukaryotes (2–4). Although genes encoding such ACCases appear to be distributed in a small group of bacteria (Supplementary Figure S1), they offer an important clue to how ACCases function and evolve, which is currently under investigation. The *accR* and *accS* genes, both of which are from single-gene operons, are divergent from each other, constitute a canonical regulatory gene organization pattern for LTTRs (38,39). In this type of genetic systems, the regulator often is subject to negative autoregulation, preventing overexpression (39,65). However, AccR is not the case. Given that forced expression of *accS* could fully correct the growth defect resulting from the AccR loss, it appears that AccR activates *accS* transcription only. Similar to some other ‘canonical’ LTTRs (39,65), in the absence of an effector, AccR binds to both RBS and ABS(II) (Figure 3C–E). Despite this, there seem to be some differences in activation mechanism between AccR and other canonical LTTRs. According to the working model of canonical LTTRs, the LTTR-effector complex binds to the ABS(I) site and interacts with RNA polymerase, leading to transcription initiation. However, ABS(I) of AccR is mutable, implying that ABS(I) may not be critically involved in activation.

Given that ACCases convert acetyl-CoA to malonyl-CoA and AccR acts as an activator, it is logical to propose that the most likely effector for AccR is acetyl-CoA. Clearly, the data obtained from the analyses carried out *in vivo*, *in vitro* and *in silico* support this notion. Among putative effectors under test, acetate, alone or with pantotheate together, were found to stimulate *accS* expression, which is dependent on AccR. In line with this, the direct interaction between AccR and acetyl-CoA was detected with ITC and MST although the binding appears to be weak and/or unstable. The structural data reveal that the effector-binding pocket of AccR in a EBD monomer is significantly larger than those observed from two other LTTRs that have a highest structural similarity. Although this pocket is apparently more spacious for acetyl-CoA, MD analysis suggests that if acetyl-CoA is the co-effector, the pocket is the likely interaction site. This gains support from the mutational analysis of residues predicted to be critical for formation of the pocket, which are found to be important for not only regulatory activity but also responsiveness to acetyl-CoA.

Since the interaction is unstable, we speculate the presence of an effecting molecule in addition to acetyl-CoA. This is not unprecedented. NdhR of cyanobacteria interacts with both 2-phosphoglycolate (2-PG) and 2-oxoglutarate (2-OG) to coordinate carbon and nitrogen metabolism (66). The binding of these effectors to the regulator is at different sites in an exclusive manner. AphB from *Vibrio* species, which is highly similar to AccR in structure (Supplementary Figure S5B and D), contains two effector-binding pockets resembling those of AccR. Biochemical screening identified a synthetic agent, BP-15, to be able to bind to the pocket located at the dimerization interface (51). In contrast, despite intensive studies for more than two decades (67,68), small molecules, either natural or artificial, that specifically bind in the primary effector-binding pocket of AphB located at the interface of RD-I and RD-II as in other LTTRs remain elusive. It therefore seems that the binding of the ligands to these two pockets may be synergistic, requiring both for forming a stable ligand-regulator complex. Furthermore, the possibility that the primary pocket of AccR may interact with a ligand that is of the right size and is derived from acetyl-CoA could not be ruled out.

AccS and its homologs are only present in *Shewanella* and a small number of genera in γ-proteobacteria. In addition to the divergent arrangement of *accR* and *accS*, in many of these organisms another LTTR is present in the same direction on the chromosome, if not next to, in proximity of the *accS* gene. However, the sequence identity between this LTTR and AccR is 30% only, even not among top five homologous LTTRs of AccR. It is well known that LTTRs are structurally similar, especially those using small molecules as effectors, but they are generally modestly conserved in sequence identity (∼30%), even for those sharing the same activating mechanisms, such as oxidative stress responding OxyRs of *S. oneidensis* and *E. coli* (69). Thus, we do not yet know whether this LTTR mediates *accS* transcription. Nevertheless, given that both bacteria and archaea are equipped a large number of LTTRs, let alone numerous single-component transcriptional regulators of other families (TetR, ArsR, MerR, MgrA, to name a few) (39,70), we imagine that transcriptional regulators that directly control transcription of genes encoding ACCase components in prokaryotes having other types of ACCases likely exist. Efforts to identify them are underway.

## Supplementary Material

gkae960_Supplemental_File

## Data Availability

All data are incorporated into the article and its online supplementary material. The structure coordinates have been deposited in the Protein Data Bank (PDB ID: 9BCE).
