# Supplementary material for: A bacterial transcription activator dedicated to the expression of the enzyme catalyzing the first committed step in fatty acid biosynthesis
Source: Nucleic Acids Res. 2024 Oct 30;52(21):12930–44. doi: 10.1093/nar/gkae960 (PMC11602165; doi:10.1093/nar/gkae960)
Supplement: gkae960_Supplemental_File [file gkae960_supplemental_file.pdf]

Supporting Information for

A bacterial transcriptional activator dedicated for expression of the enzyme that catalyzes the first committed step in fatty acid biosynthesis

Yuanyou Xu,[1#] Zihan Lin,[2#] Jiyuan Hou,[1] Kai Ye,[2] Sirui Han,[1] Yuxuan Liang,[1] Huihui Liang,[1] Shihua Wu,[1] Yizhi J Tao,[2*] and Haichun Gao[1,2*]

This PDF file includes:

Tables S1 to S2
Figures S1 to S9

Table S1. Strains and plasmids used in this study

Strain or plasmid	description	Source/reference
Strains		
E.coli		
DH5α	Host strain for plasmids	Lab stock
WM3064	Donor strain for conjugation; Δ*dapA*	W. Metcalf, UIUC
BL21(DE3)	Expression host	Lab stock
BTH101	Reporter strain for Bacterial Adenylate Cyclase Two-Hybrid (BACTH)	EUROMEDEX
ER2738	Host strain for intermediate construction in BACTH	Lab stock
S. oneidensis		
MR-1	Wild type	Lab stock
HG0839	Δ*accR* derived from MR-1	This study
SO-X4	A Spontaneous mutant from MR-1	This study
Plasmids		
pHGM01	Ap^r Gm^r Cm^r suicide vector	(20)
pHGI01	Km^r, integrative *lacZ* reporter system	(22)
pHGEN-P*tac*	IPTG-inducible P*tac* expression vector	(21)
pET28a	Ap^r expression vector	Lab stock
pKT25	Km^r, IPTG-inducible co-expression with T25 fragment vector	EUROMEDEX
pUT18C	Am^r, IPTG-inducible co-expression with T18 fragment vector	EUROMEDEX
pKT25-zip	GCN4 and T25 fragment fusion proteins, positive control for BACTH	EUROMEDEX
pUT18C-zip	GCN4 and T18 fragment fusion proteins, positive control for BACTH	EUROMEDEX
pHGEN-P*tac*-AccR^{His6}	inducible expression of AccR^{His6}	This study
pHGEN-P*tac*-AccR-EBD^{His6}	inducible expression of AccR-EBD^{His6}	This study
pHGEN-P*tac*-AccS	inducible expression of AccS	This study
pHGEN-P*tac*-EcAcc	inducible expression of *E. coli* AccA-AccD-AccBC	This study
pHGEN-P*tac*-AccR^{v}	inducible expression of AccR variants	This study
pKT25-*AccR*	AccR and T25 fusion proteins expression	This study
pKT25-*AccR*^{S103A}	AccR^{E105A} and T25 fusion proteins expression	This study
pKT25-*AccR*^{E105A}	AccR^{E105A} and T25 fusion proteins expression	This study
pUT18C-*AccR*	AccR and T18 fusion proteins expression	This study
pUT18C-*AccR*^{S103A}	AccR^{E105A} and T18 fusion proteins expression	This study
pUT18C-*AccR*^{E105A}	AccR^{E105A} and T18 fusion proteins expression	This study
pHGI01-PaccS	For measuring activity of AccS promoter	This study
pHGI01-PaccS^{v}	For measuring activity of AccS promoter variants	This study
pET-28a-AccR^{His6}	Inducible expression of AccR^{His6} in *E. coli*	This study
pET-28a-eGFP-AccR-EBD^{His6}	Inducible expression of eGFP-AccR-EBD^{His6} in *E. coli*	This study
pET-28a-AccR-EBD^{His6}	Inducible expression of AccR-EBD^{His6} in *E. coli*	This study
pET-28a-AccR$^{K36E\text{-}His6}$	Inducible expression of AccR$^{K36E\text{-}His6}$ in *E. coli*	This study
pET-28a-AccR$^{K36Q\text{-}His6}$	Inducible expression of AccR$^{K36Q\text{-}His6}$ in *E. coli*	This study

Ap^r, apramycin resistance; Gm^r, gentamicin resistance; Cm^r, chloramphenicol resistance; Km^r, kanamycin resistance.

Table S2. Data collection, phasing and refinement statistics

	AccR Se-SAD
**Data collection**	
Space group	C2
Cell dimensions	
a, *b*, *c* (Å)	90.0, 43.0, 112.1
α, β, γ (°)	90.0, 104.2, 90.0
Wavelength (Å)	0.97856
Resolution (Å)	50 – 1.402
R_{sym} or R_{merge} (%)	10.08
$I / \sigma I$	12.41/1.92
Completeness (%)	94.81
Redundancy	7.3
**Refinement**	
Resolution (Å)	39.01 – 1.402 (1.452 – 1.402)
No. reflections	134465
R_{work} / R_{free} (%)	16.98/19.58
No. atoms	
Protein	3268
Ligand/ion	0
Water	502
B-factors	
Protein	17.67
Ligand/ion	N/A
Water	29.03
R.m.s deviations	
Bond lengths (Å)	0.008
Bond angles (°)	1.026
Ramachandran statistics	
Favored (%)	97.81
Allowed (%)	2.19
Outlier (%)	0.00

*Number of xtals for each structure should be noted in footnote. *Values in parentheses are for highest-resolution shell.

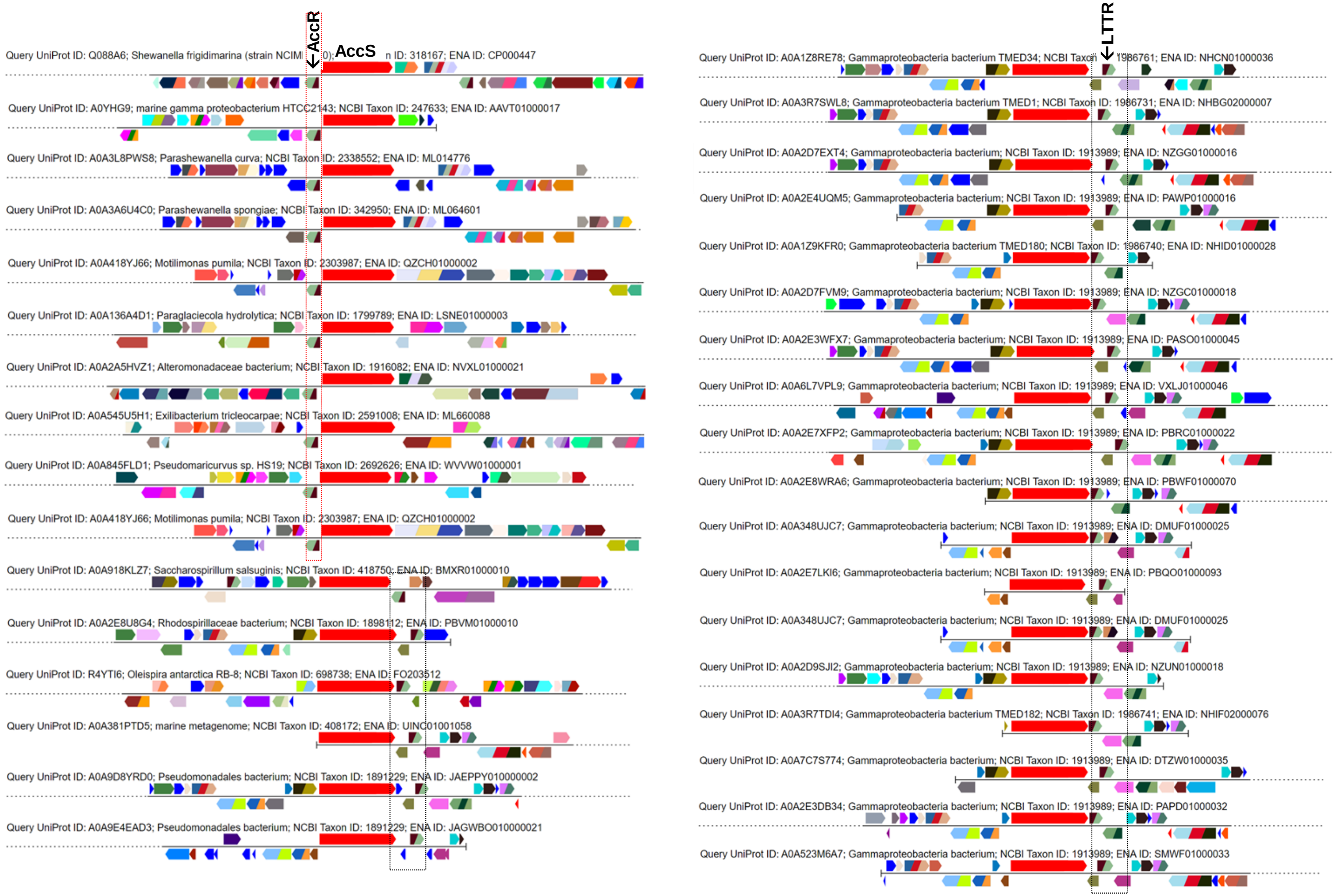

Fig. S1 Distribution of a LTTR gene linked with *accS* on the chromosome. The *accR*-*accS* organization is conserved in *Shewanella* and is present in some other genera in γ-proteobacteria. In addition to this, the *accS*-LTTR organization is also present, which is more widely distributed in γ-proteobacteria.

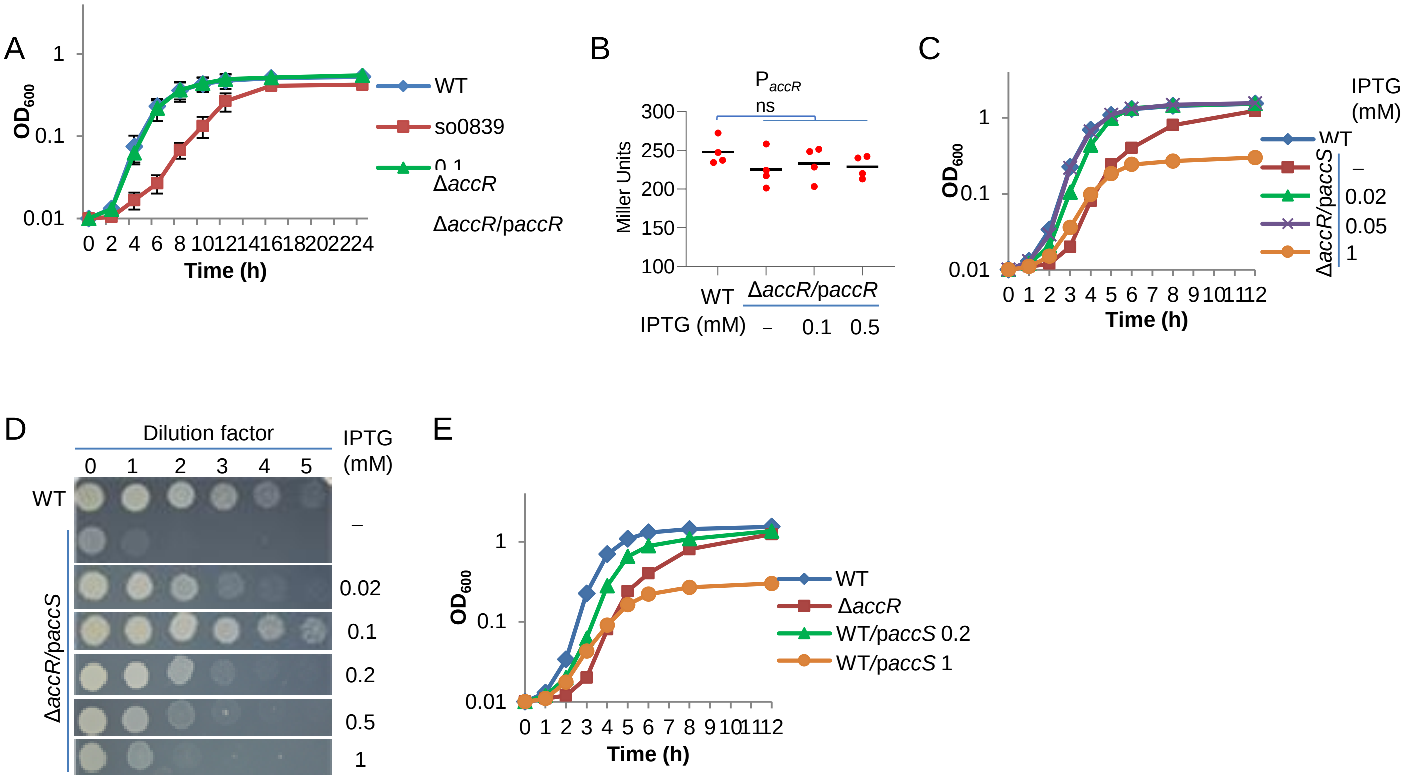

Fig. S2 Reduced expression of AccS underlies growth defect of SO-X4. (A) Growth in defined medium MS. p*accR*, a copy of the *accR* gene was expressed *in trans* for complementation under control of IPTG inducible promoter P*tac*, with 0.2 mM IPTG. (B) Activity of the *accR* promoter in cells grown in LB. p*accR*, a copy of the *accR* gene was expressed *in trans* for complementation. (C) Effects of *S. oneidensis accS* expressed to varying levels on growth of Δ*accR*. (D) Effects of *S. oneidensis accS* expressed to varying levels on growth of Δ*accR* on LB agar plates. (E) Effects of *S. oneidensis accS* expressed to varying levels on growth of WT.

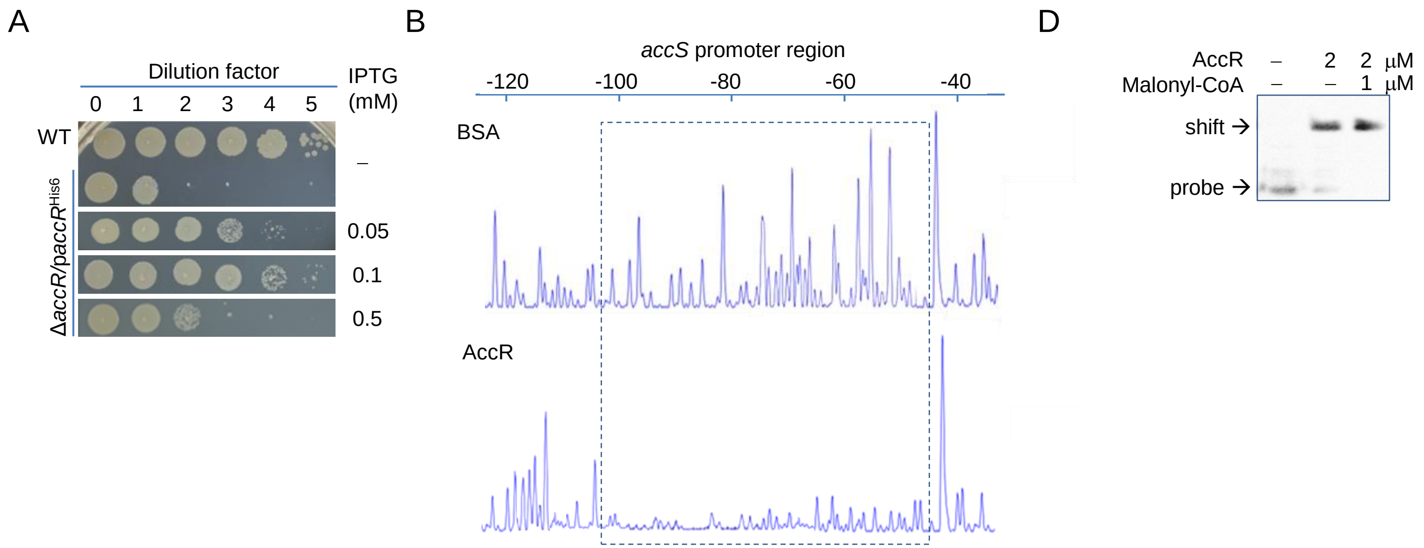

C

```
                        RBS                          ABS                  ABS
AccR-accS:  CCACTGTCTCATATTTGGAACATAGCGTCTTATGTTGCAGTTTAATGGCATTTGGCAGGATAACAAAGTTATATT
CatR-catB:  CCATCAGACCTCCAGGGTATGGTGGAGATTCATTCGATATTGGACGGCTATCAGGGTCTCGCGCAATACCTTGA
BenM-benA:  TAAAAATACTCCATAGGTATTTTATTATACAAATAAATGTGTTTGAACTTATTAAAACATTCTTTTAAGGTATAAA
ClcR-clcA:  AAGCCATACCGATCCCGTATTGCAAAGGCTAAAAAAAGGTATTGGACCGCATGACACGCGAATCTTAGCATTCAT
Consensus:        T     N11     A
```

Fig. S3 AccR regulates *accS* transcription. (A) Effects of *S. oneidensis* His$_6$-tagged AccR expressed to varying levels on growth of Δ*accR* on LB agar plates. (B) DNase I footprinting assay of AccR binding to the promoter region of *accS*. The electropherograms represent control DNA with BSA (bovine serum albumin) in the upper panels and footprints with of AccR in the lower panels. The black dash box depicts the region protected from DNase I digestion by AccR. The *accS* promoter region is numbered the same as in Fig. 4E. (C) Comparison of DNA motifs of four LTTRs that control a divergent promoter. (D) Effects of exogenous malonyl-CoA on binding of AccR to P$_{accS}$ (P$_{129}$) analyzed by EMSA.

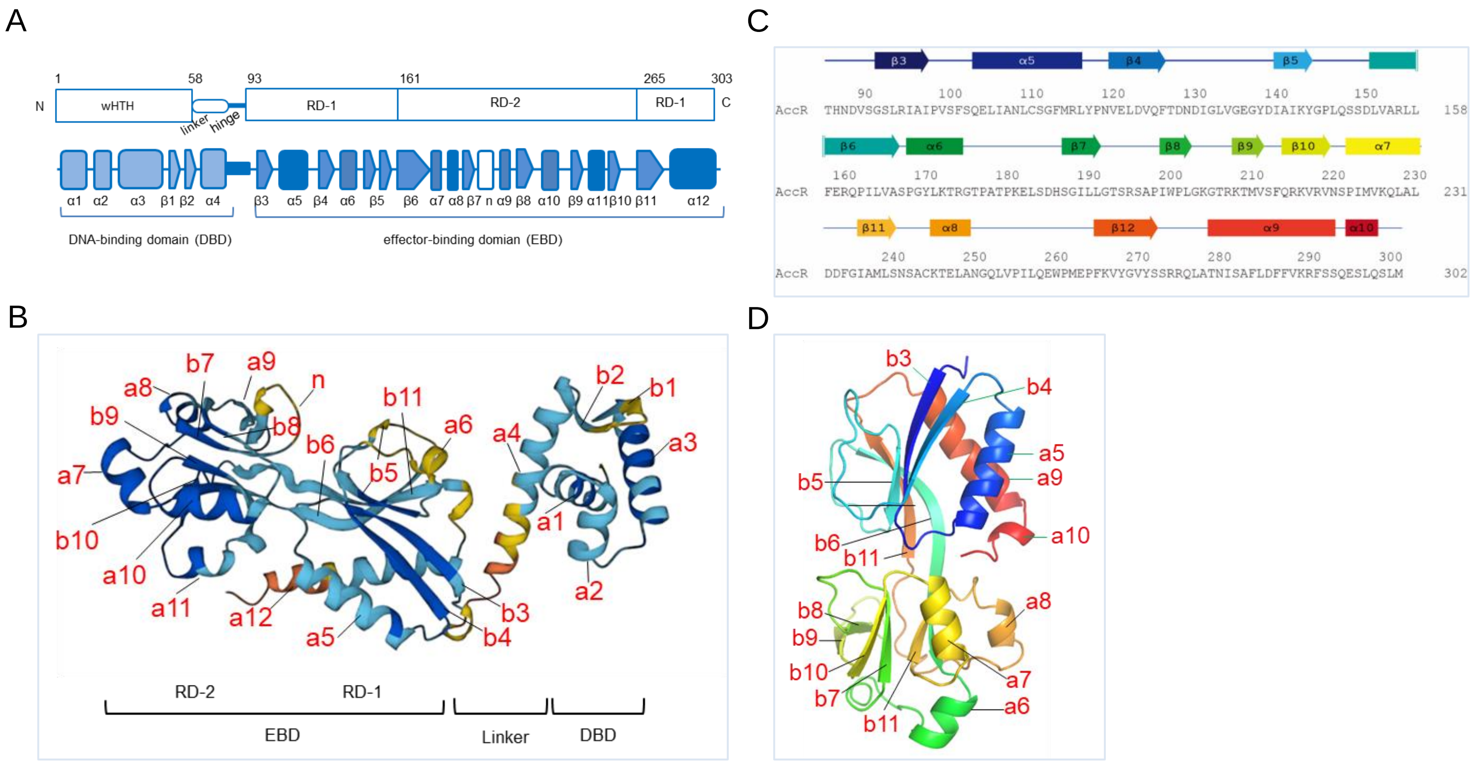

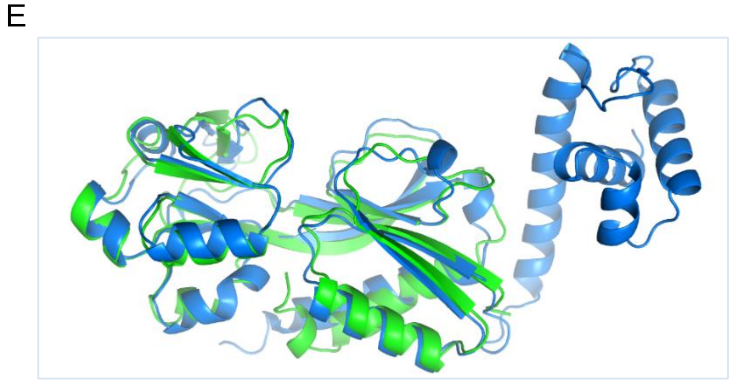

Fig S4. Domain architecture and secondary structure elements of AccR. (A) Domain architecture and secondary structure elements of AccR predicted by Alphafold2 (AFDB ID: AF-Q8EIK0-F1). (B) Ribbon diagram of the predicted structure of AccR. The molecule is colored by the pLDDT values with high and low confidence regions shown in blue and red, respectively. (C) Domain architecture and secondary structure elements of AccR EBD based on the structure solved in the study with ribbon diagram of the structure shown in (D). (E) Superimposition of AccR structures predicted (blue) and experimentally solved (green, only EBD).

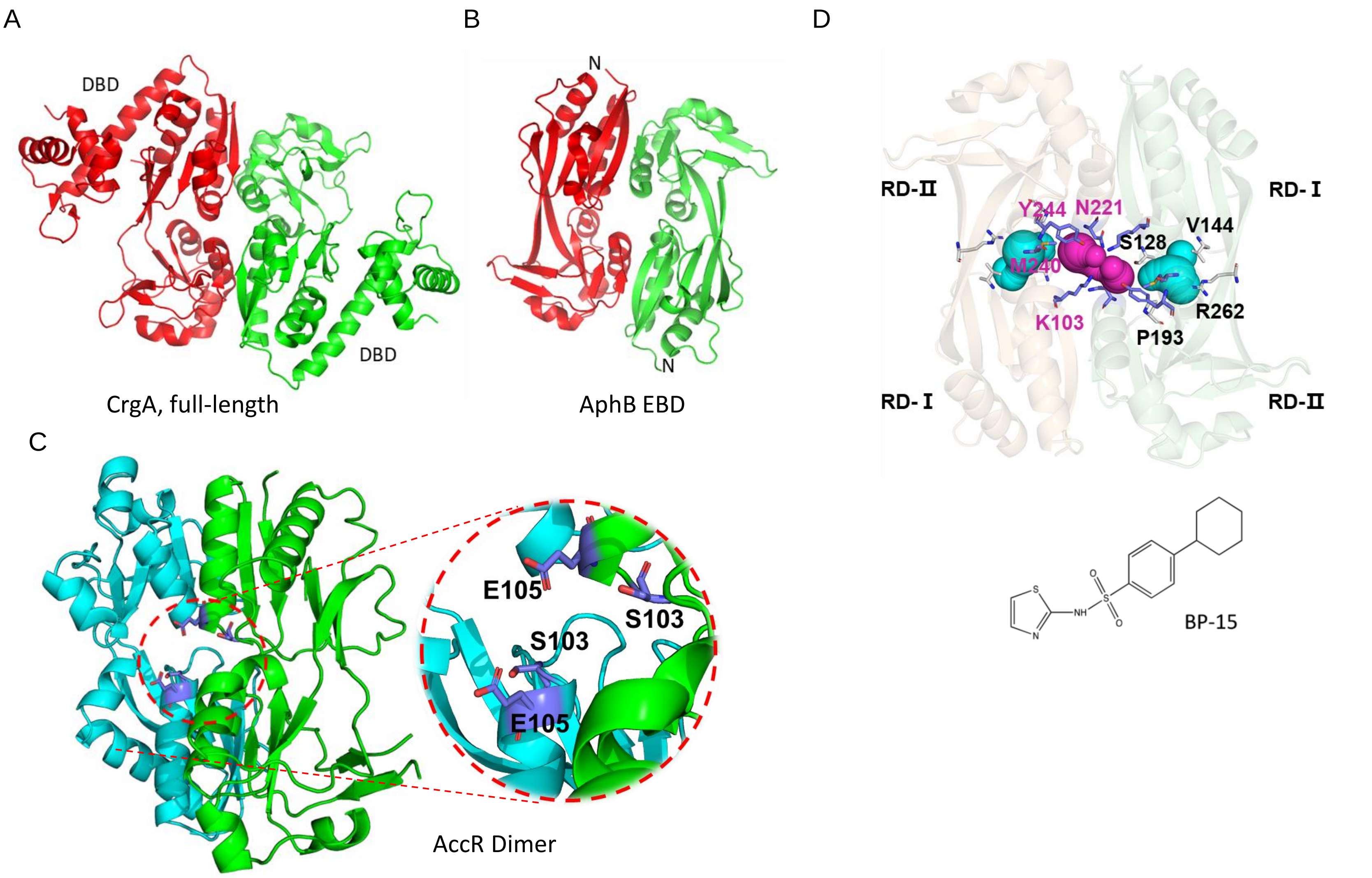

Fig S5. AccR EBD dimer. (A) CrgA, full-length. The two DBD domains from the two subunits are labeled. (B) AphB EBD dimer. (C) An additional putative effector-binding pocket with key residues. (D) Pockets identified in the *V. cholerae* AphB dimer (PDB: 5X0O). AphB has been suggested to have two effector-binding pockets. The conventional one present in each monomer, as observed in other LTTRs mentioned in this study, are colored cyan. The effector binding to this pocket remains unknown. The other in magenta is located in the interface of the dimer. Although its natural ligand is yet to be identified, BP-15 interacts with this pocket.

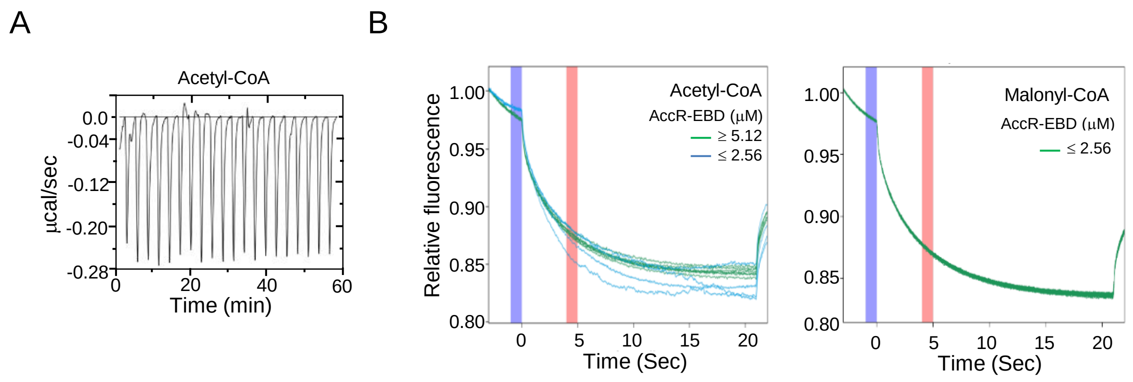

Fig S6. Thermophoretic analysis of the interaction of AccR with acetyl-CoA and malonyl-CoA. (A) The ITC binding isotherm of the AccR EBD titrated into acetyl-CoA or malonyl-CoA at 298K in Tris buffer. Raw data from titration of consecutive 10 μl injection of acetyl-CoA (1.8 mM) into AccR EBD (36 μM), represented as the heat change (cal/s) upon injection over time. Positive control (Bottom) included in the assay to validate the experimental system. (B) Microscale thermophoresis (MST) profiles of the AccR EBD and acetyl-CoA or malonyl-CoA interaction. Fcold (blue region) at 0 s represents the cooled state, and Fhot (red region) at 5 s represents the heated state during thermophoresis. eGFP-AccR^{EBD} expressed and purified from *E. coli was* used for the experiment. Protein concentrations (μM): 0.16, 0.32, 0.64. 1.28, 2.56, 5.12, 10.3, and 20.6.

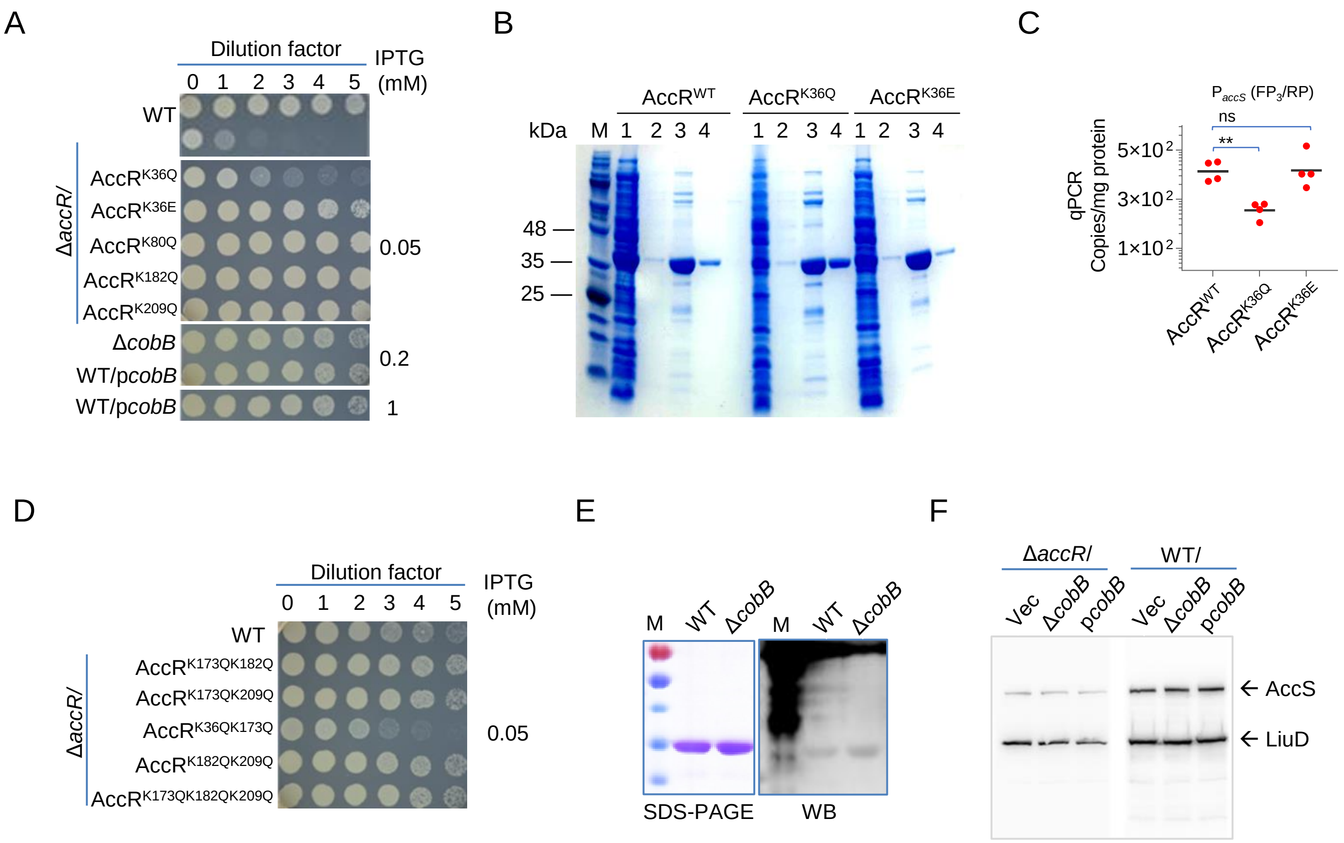

Fig S7. AccR functions independent of acetylation. (A) Growth of Δ*accR* expressing AccR acetylation variants. Acetylation residues were mutated individually and in combination (refer to Fig. 6D, 6E). (B) Purification of His-tagged AccR^{WT}, AccR^{K173Q} and AccR^{K173Q}. M, marker; 1, starting material; 2, wash buffer; 3, pooled fractions after Ni-NTA step, 4, purified protein after SEC. (C) EMSA analysis of effect of acetylation at K36 on binding of AccR to P_{accS} (P_{129}). (D) qPCR analysis of effect of acetylation at K36 on binding of AccR to P_{accS} (P_{129}). DNA extracted from cells grown to the mid-exponential phase supplemented with acetate was quantified with primers RP and FP_3. (E) Growth of Δ*accR* expressing AccR acetylation variants as in (A). (E) Impacts of CobB on AccR acetylation. Western blotting (WB) of AccR with acetylated-lysine antibody. (F) Impacts of CobB on AccS levels. Endogenous biotinylated proteins identified by Western blot. IPTG, 0.5 mM.

A B

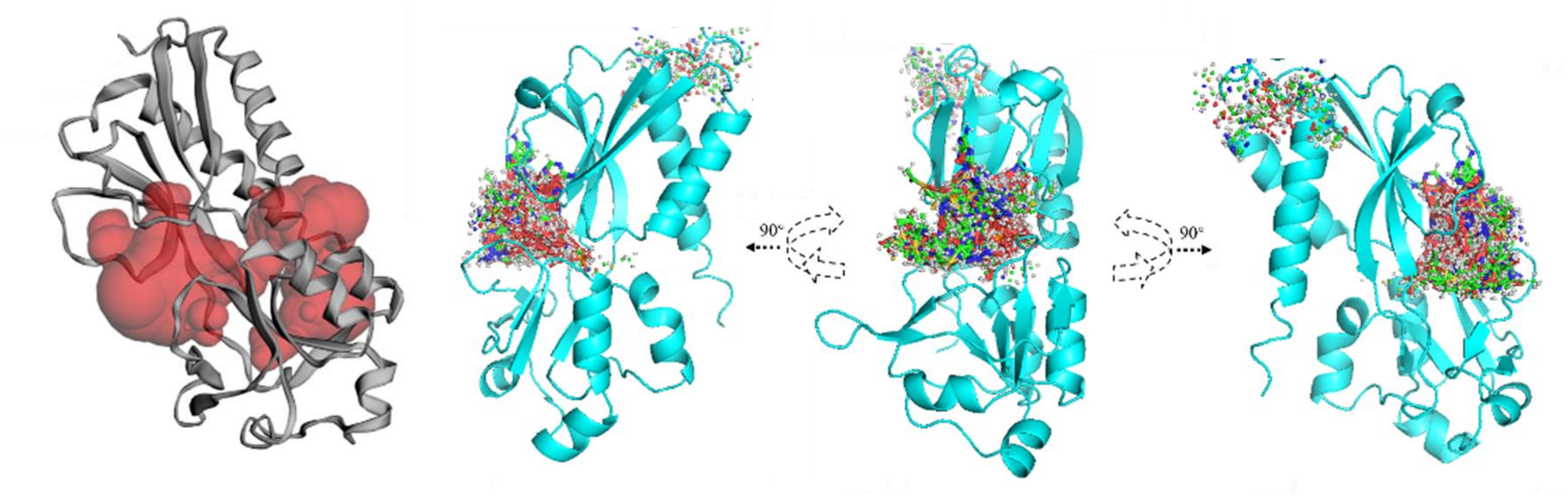

Fig. S8 Putative binding pocket identified by in silico analysis. (A) The putative effector pocket predicted by CASTp is shown with the potential pocket highlighted in red. (B) Discovery of the pocket by SwissDock, all poses of acetyl-CoA are depicted in the figure (colorful dots represent ligand atoms, and partial ligand bonds are hidden).

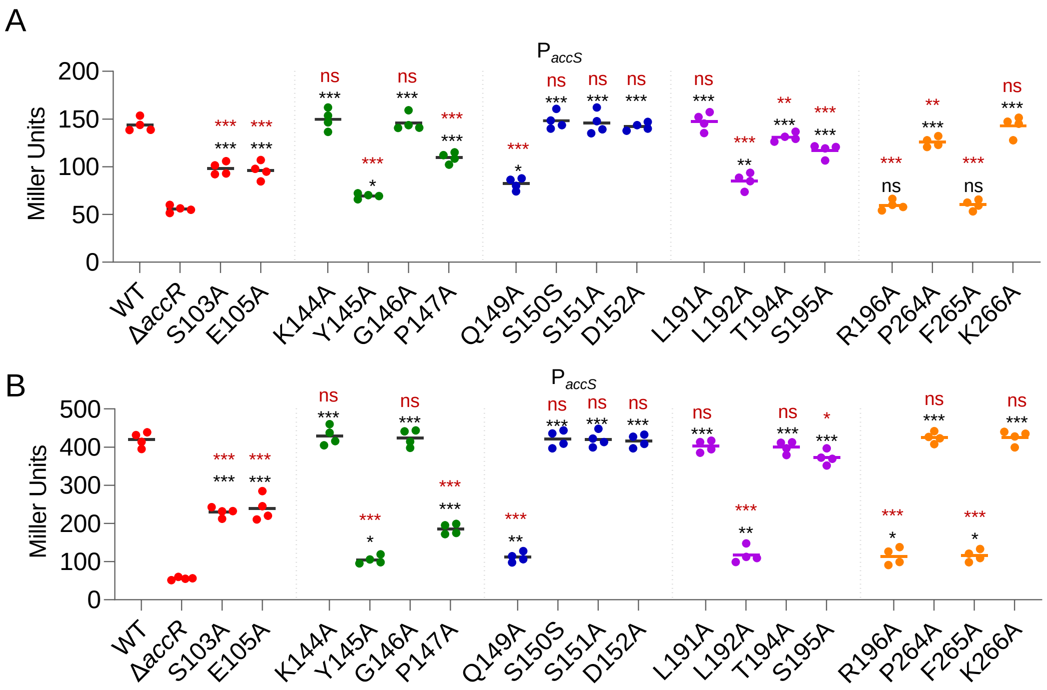

Fig. S9 Both putative effector-binding pockets are functionally important. Alanine scanning was carried out on residues predicted to be involved in formation of two effector-binding pockets. (A) In Δ*accR,* each AccR variant was expressed with 0.2 mM IPTG and expression of *accS* was measured. The experiment was carried out the same as in Fig. 2B. (B) The same experiment as (A) with addition of 5 mM acetate. Statistics analysis was applied for the values compared to those of WT (upper) and Δ*accR* (lower), ns, not significant; *, p < 0.05; **, p < 0.01; ***, p < 0.001.
